# Association of childhood and adolescence obesity with incidence and mortality of adulthood cancers. A systematic review and meta-analysis

**DOI:** 10.3389/fendo.2023.1069164

**Published:** 2023-01-19

**Authors:** Nami Mohammadian Khonsari, Ehsan Shahrestanaki, Amir Ehsani, Sara Asadi, Leily Sokoty, Sahar Mohammadpoor Nami, Benyamin Hakak-Zargar, Mostafa Qorbani

**Affiliations:** ^1^ Non-Communicable Diseases Research Center, Alborz University of Medical Sciences, Karaj, Iran; ^2^ School of Medicine, Iran University of Medical Sciences, Tehran, Iran; ^3^ Western Sydney University, Translational Health Research Institute, Sydney, NSW, Australia; ^4^ Social Determinants of Health Research Center, Alborz University of Medical Sciences, Karaj, Iran; ^5^ School of Medicine, Faculty of Health, Deakin University, Geelong, VIC, Australia; ^6^ Endocrinology and Metabolism Research Center, Endocrinology and Metabolism Clinical Sciences Institute, Tehran University of Medical Sciences, Tehran, Iran

**Keywords:** cancer, childhood, adolescence, obesity, malignancy, adulthood

## Abstract

**Background:**

Prevalence and subsequent conditions of childhood and adolescent obesity are increasing. It has been seen that obesity in youth is associated with adulthood cancer. This systematic review and meta-analysis aimed to determine the pooled association of childhood obesity with cancers in adulthood.

**Methods:**

In this systematic review, international electronic databases such as Scopus, PubMed, Web of Science, and EMBASE were searched using relevant keywords until February 2022. All Cohort studies assessing the association of childhood and adolescent obesity (under 18 years old) with the incidence and mortality of all types of cancers were included. Two independent reviewers screened and carried out the quality assessment of included studies. Between-studies heterogeneity was assessed using the I squared and Cochran’s Q tests. Random/fixed-effect meta-analyses were used to pool the appropriate effect sizes (Hazard ratios (HR)).

**Results:**

Overall, 46 studies were found to be relevant and were included in this study. Based on the random-effects model meta-analysis, childhood obesity increased the hazard of cancer incidence and mortality in adulthood by 33% (HR: 1.33, 95%CI (1.25, 1.41)) and by 28% (HR: 1.28, 95%CI (1.13, 1.42)), respectively. In the subgroups meta-analysis, the HR of childhood obesity and adulthood cancer incidence mortality in women was higher than in men (HR=1.39, 95%CI (1.25, 1.53) vs HR= 1.20, 95%CI (1.07, 1.32)) and (HR= 1.40, 95%CI (1.10, 1.69) vs HR=1.20, 95%CI (1.04, 1.36)) respectively.

**Conclusion:**

This study found that obesity in childhood and adolescence is associated with a significant increase in the incidence and mortality of cancers in adulthood. Prevention of childhood obesity, in addition to its short-term beneficial effects, can reduce the burden of cancer in adulthood. The data sets of this study are present in the Tables of the current manuscript. Moreover this study was registered online in PROSPERO (registration code: CRD42022331958).

**Systemic review registration:**

https://www.crd.york.ac.uk/Prospero/, identifier CRD42022331958.

## Background

Obesity is a condition notorious for its associated morbidity and mortality (1); and plays a part in the prevalence of the leading causes of death and disability worldwide [e.g. Hypertension, diabetes, cancer and so on (1)]. The association between obesity and many of these conditions are well-studied and established; however, its association with cancer is intriguing and complex.

Cancer has resulted in millions of deaths and morbidities throughout the years, thus earning its place among the most significant causes of mortality and morbidities worldwide (2). Despite impressive breakthroughs in Cancer treatment in the past decades, which reduced various cancers’ morbidities and mortalities, it still is a major foe of human health (3). Hence, due to cancers’ resilience to treatment, researchers have also studied the genetics and epigenetics of cancer to prevent it. Although cancer genetic factors are primarily un-adjustable, some epigenetic factors are changeable, and interventions in environmental and epigenetic factors have been introduced to reduce cancer risk. Nevertheless, implementing these interventions requires a thorough knowledge of the epigenetic risk factors of cancer (4, 5).

Similar to epigenetic factors, obesity is a modifiable cancer risk factor (6). Numerous studies have shown that obesity is a significant risk factor for cancer incidence (7–10); moreover, some have stated that it has a protective effect against certain malignancies (11). To better illustrate this association, cohort studies have been designed to determine the association between obesity and cancer (12). Furthermore, preventive measures are being carried out; however, despite our efforts, within the past few decades, the prevalence of both obesity and cancer has been increasing dramatically (13, 14). This increasing trend has also been seen in children and adolescents (15–17). This fact is worrisome since cancer and obesity are associated, and obesity is a chronic condition that tends to persist (18); the longer it persists, the longer the obese individual is at risk of its associated conditions such as cancer (19). Hence obesity in children and adolescents is of particular importance. Nonetheless, despite this importance, few systematic reviews have evaluated the association between obesity in children and adolescents and cancer in adulthood; these studies either are based on a single malignancy incidence (20, 21), or the studies included adults in their analysis as well (22). Hence unlike previous studies, this study aimed to assess the association of obesity in children and adolescents with the incidence of various types of malignancies and malignancy-related mortality.

## Methods

In conducting this study, we adopted the Preferred Reporting Items for Systematic Review and Meta-Analysis (PRISMA) guidelines.

### Search strategy

A systematic search of the available literature was conducted across electronic databases (Scopus, PubMed, Web of Science, and EMBASE till the end of February 2022. One of the researchers conducted the search, and another researcher reviewed the results. The searched terms were “cancer”, “ malignancy”, “neoplasm”, “obesity” and their equivalent terms (based on MesH terms). The search strategy is presented in full within the **supplementary Table 1**.

### Eligibility criteria and selection study

All Cohort studies with an English full text assessing the relationship between obesity in children and adolescents and cancers in adulthood were included in this study. All studies must have represented the targeted population in exposure (obesity in children and adolescents) and outcome (cancer incidence and/or mortality in adults). Studies must have appropriately determined cancer incidence and mortality and had proper documentation and measures regarding childhood and adolescent weight, height, and BMI. Studies without the aforementioned criteria were excluded (e.g. studies based on self-reported BMI values, etc.)

After duplicate removal with EndNote X9, two investigators independently assessed the titles and abstracts and, lastly, the full texts of the remaining articles. Moreover, the reference lists of the included studies were hand-searched to find other relevant studies. Discrepancies were referred to the third investigator for resolution.

### Data extraction strategy

A pre-designed data extraction data sheet consisting of the first author’s name, year of the study, age range or mean sex, the number of participants, type of malignancy, obesity definition, and measure (qualitative or quantitative) was used. Hazard ratios (HR), Odds ratios (OR), or Risk ratio (RR) (otherwise known as relative risk) alongside their 95% confidence interval (CI) as the effect size and studies’ adjustments for potential confounders were recorded. Two researchers extracted the data, and possible discrepancies were referred to the third researcher for resolution.

### Quality assessment (QA)

The Newcastle-Ottawa Scale for cohort studies was used for quality assessment. We scored the studies based on selection, outcome, and comparability with this seven-item scale. The total score, which is the sum of the scores of its items, ranges from 0 to 9. We categorized the studies based on their scores (below 5: unsatisfactory, 5-7: satisfactory, and 8-9: high-quality studies). Two investigators scored the studies independently, and discrepancies were referred to the third researcher for resolution.

### Statistical analysis

The I squared, and Cochran’s Q tests were adapted to assess heterogeneity between studies. If the heterogeneity P-value was less than 0.1, a random-effect model (DerSimonian–Laird) was used to pool the effect sizes. Otherwise, a fixed model was adopted. Meta-analysis was performed for outcomes with at least two records with identical measurements. Only the HRs and RRs were combined and pooled in the Meta-analysis (23, 24).

Binary BMI measurement was considered as the highest to the normal measure based on the study reports (highest quartile or quantile or obese individuals to the normal or non-obese population). Continuous BMI measure was considered as a one unit change in BMI Z-score or standard deviation (SD). The HRs or RRs of studies with two and above identical measures were pooled, and then a singular HR or RR for that study was used in the meta-analysis.

Sub-group analysis was performed according to cancer types and sex. Egger’s test for each malignancy determined the level of publication bias. In cases where publication bias was present, trim fill analysis was performed to impute the missing data.

STATA (Stata Corporation, College Station, Texas, USA) version 17 was used for data analyses.

## Results

### Search results

**Figure 1:**
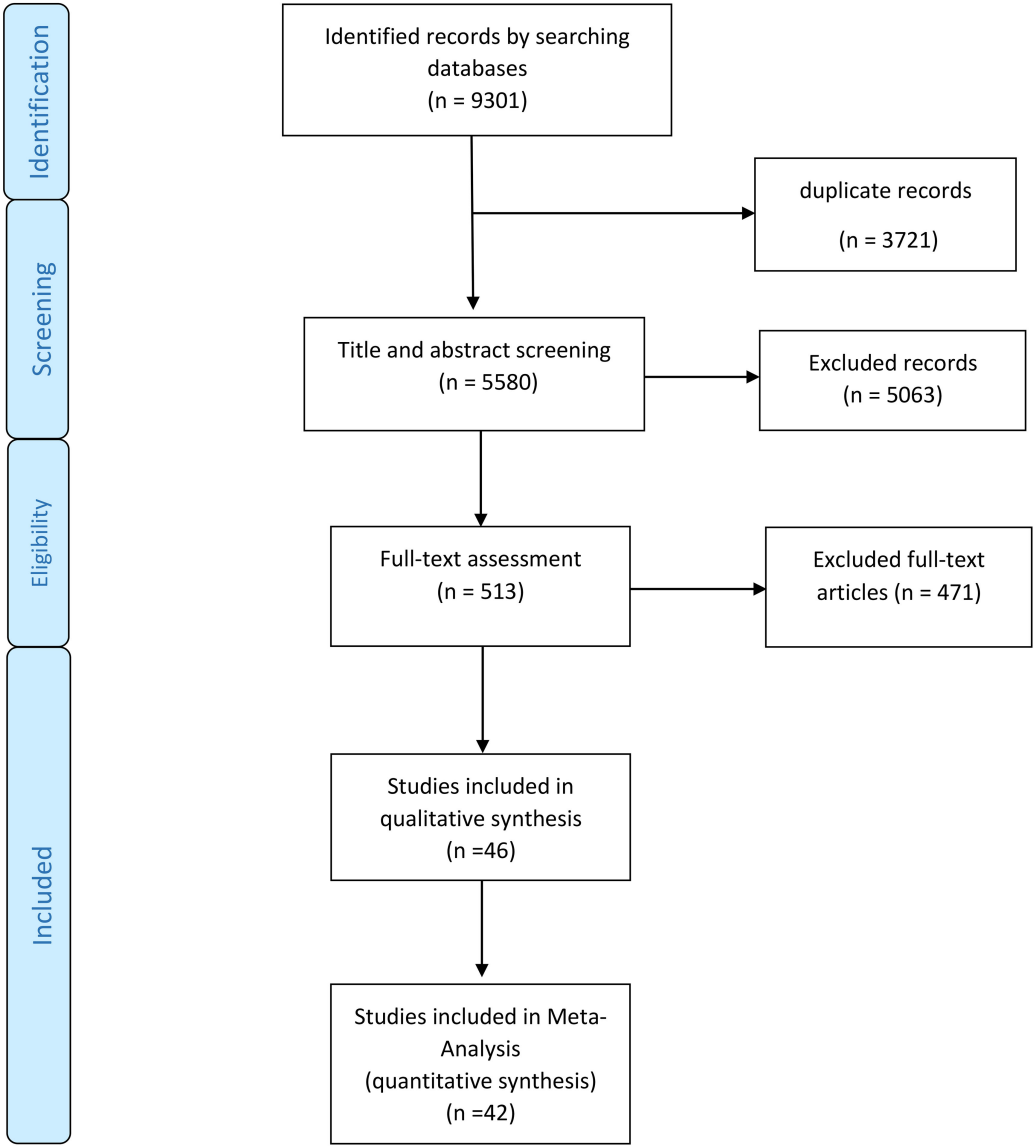
Studies search, review and analysis flowchart.

Of the initial 9301 studies found in the initial search (plus hand-searched studies), 3721 were duplicates, the titles or title and abstracts of the remaining 5580 articles were evaluated, and 5063 were found to be irrelevant to this study. The full texts of the 513 remained articles were assessed, and 471 were excluded. Lastly, 46 articles remained in the study and were used for qualitative, and 42 were included in the quantitative data synthesis. The entire illustration of this process can be seen in **Figure 1**.

The majority of the found studies were conducted in Europe (Denmark, Sweden, Norway, the United Kingdom (UK), and Finland). Other studied countries included Israel, the USA, and Australia. Denmark and Israel had the most studies (17 studies); USA, Finland and Australia had the least number of studies, with only one study assessing them all together.

The largest sample size was from Israel, with 19567635 participants. The smallest was from the UK, with an overall participant number of 6842. Of all the 46 studies,4 focused on cancer mortality/survival; the other 42 assessed cancer incidence. Moreover, out of these 42 articles, most studies assessed gastrointestinal (GI) related malignancies (18 studies) (Esophagus, Stomach, Liver, Pancreas, Colorectal). These data, alongside other primary findings, are shown in **Supplementary Table 2**. Regarding quality assessment, 4 of the included studies scored 7 (satisfactory), and the other 42 scored 8 or 9 (high quality); the scorings can be found in the **Supplementary Table 3**.

### Qualitative synthesis

The association of childhood and adolescence obesity-related indices with cancers in adulthood in included studies is shown in **Supplementary Table 4**. Nearly half of the reported effect sizes are statistically significant. The reported HR ranges for the association of childhood and adolescent obesity and cancer incidence in adulthood based on cancer types were as follows (HR, (95%CI)): Prostate cancer: 1.02 (0.96-1.09) to 1.17 (0.97-1.41), endometrial cancer: 1.00 (0.78-1.27) to 1.39 (1.22-1.58), ovarian cancer: 0.75 (0.48-1.16) to 3.71 (1.63-8.46), Breast cancer: 0.69 (0.48-1.00) to 1.05 (0.95-1.14) in females and 2.01 (1.14-3.54) to 4.97 (2.14-11.53) in males, liver cancer: 1.15 (0.95-1.40) to 3.59 (1.85-6.99), renal cell carcinoma: 1.45 (0.63-3.34) to 2.87 (1.32-6.25), Testicular Cancer: 0.88 (0.46-1.67) to 1.29 (0.84-1.98), Hematologic malignancies: 0.89 (0.60-1.32) to 1.81 (1.13-2.92), colorectal cancer: 0.85 (0.50-1.44) to 2.62 (1.62-4.25), stomach cancer: 0.55 (0.24-1.24) to 2.91 (1.15-7.37), Esophageal cancer: 1.10 (0.94-1.29) to 3.11 (1.68-5.76), thyroid cancer: 0.99 (0.74-1.33) to 1.30 (1.08-1.55), pancreatic cancer: 0.47 (0.67-3.33) to 3.90 (1.70-8.92) and cancer mortality: 0.6 (0.3-1.4) to 2.1 (1.1-4.1). The reported effect sizes for other cancers, by sub-type (histology) of cancer, sex, age, etc., can be found in **Supplementary Table 4**

### Quantitative synthesis

The pooled association between obesity in children and adolescents and the incidence and mortality of cancers in adulthood is shown in **Table 1**. Significant heterogeneity was observed among the included studies. Based on the random effect analysis, in binary (highest (in terms of BMI, obesity, etc.) to the reference group ratio) and continuous data, obesity significantly increased the hazard of all-cause cancer incidence and mortality in adulthood in comparison with the normal weight participants by 33% (HR: 1.33, 95%CI (1.25, 1.41)) and by 28% (HR: 1.28, 95%CI (1.13, 1.42)), respectively.

**Table 1:** Meta-analysis of the association between obesity –related indices in children and adolescents with the incidence and mortality of all- cause cancer in adulthood.

	BMI	Number of studies	Pooled HRs (95% CI)	Heterogeneity assessment		
				I-squared %	Q test	P-value
Incidence						
Overall	Continuous ^1^	22	1.09 (1.06, 1.12)*	92.51	342.99	<0.001*
	Binary ^2^	29	1.33 (1.25, 1.41)*	80.92	235	<0.001*
Sex						
Male	Continuous ^1^	6	1.10 (1.05, 1.14)*	74.56	27.52	<0.001*
	Binary	16	1.39 (1.25, 1.53)*	67.35	55.13	<0.001*
Female	Continuous ^1^	9	1.06 (1.00, 1.11)*	91.37	104.29	<0.001*
	Binary ^2^	10	1.20 (1.07, 1.32)*	49.49	23.76	0.022*
Both	Continuous ^1^	11	1.11 (1.05, 1.16)*	93.64	188.64	<0.001*
	Binary ^2^	11	1.47 (1.28, 1.66)*	81.62	70.71	<0.001*
Mortality						
Overall	Binary ^2^	2	1.28 (1.13, 1.42)*	75.96	2.80	0.001*
Male	Binary ^2^	3	1.20 (1.04, 1.36)*	64.67	5.66	0.059
Female	Binary ^2^	2	1.40 (1.10, 1.69)*	82.81	11.64	0.003*

* statistically significant (P-value <0.05).

HR, hazard ratio; CI, confidence interval; BMI, Body mass index.

^1^: Continuous: one unit increase in BMI Z-score or SD, ^2^: Binary: Highest measure/normal measure.

Sex stratified results among Women and men were (HR:1.20, 95%CI (1.07, 1.32), HR:1.39, 95%CI (1.25, 1.53)) for cancer incidence and (HR:1.40, 95%CI (1.10, 1.69), HR:1.20, 95%CI (1.04, 1.36)) for cancer mortality, respectively. Also, in the continuous data (per 1 Z-score/SD increase in BMI), the hazard of cancer incidence in obese individuals significantly increased by 9% (HR:1.09, 95%CI (1.06, 1.12)); Moreover, the hazard ratio based on continuous data in both men and women were statistically significant (HR:1.10, 95%CI (1.05, 1.14)) (HR:1.06, 95%CI (1.00, 1.13) respectively).

**Figure 2:**
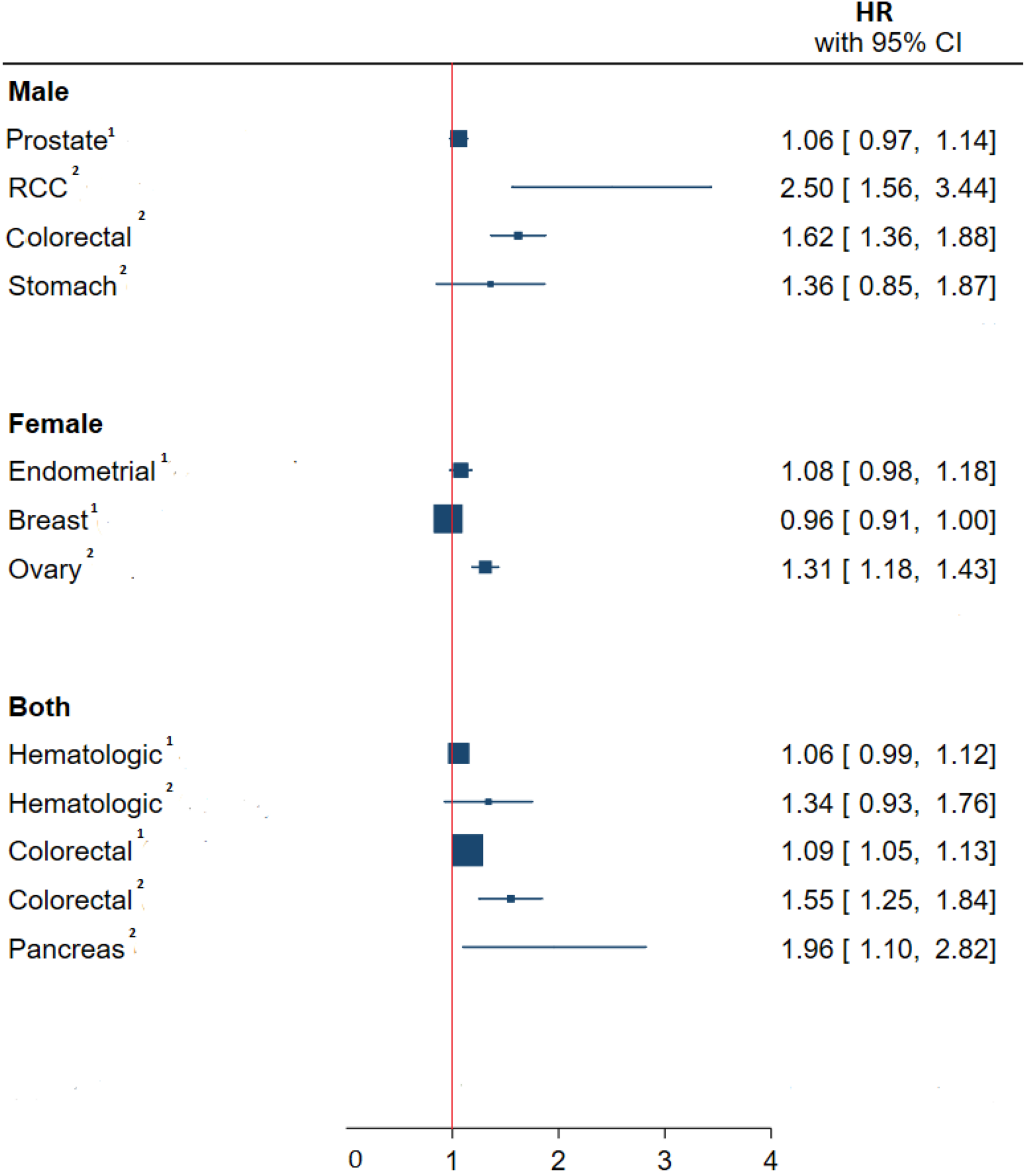
Meta-analysis of the association between obesity –related indices in children and adolescents with type of cancer in adulthood. HR, hazard ratio; CI, confidence interval; RCC, renal cell carcinoma. ^1^, Continuous, one unit increase in BMI Z-score or SD; ^2^, Binary, Highest measure/normal measure.

The pooled associations of BMI in the youth and cancers in adulthood sub-grouped by type of malignancy can be seen in **Figure 2**. The highest significant pooled hazard of cancer incidence by type in men was renal cell carcinoma (RCC) (HR: 2.50 95%CI (1.56, 3.44)) and in women was ovarian cancer (HR: 1.31, 95%CI (1.18, 1.43)). Also, in both sexes, the highest and lowest significant hazard of cancer incidence by type was observed in pancreatic (HR:1.96 95%CI (1.10,2.82) and colorectal cancer (HR:1.09 95%CI (1.05,1.13) respectively.

### Meta regression

To assess the possible causes of heterogeneity among studies meta-regression was performed for the binary and continuous overall incidence of cancer with sex, type of cancer and the country in which the study was conducted in as moderators. It was found that neither of the aforementioned variables were the underlying cause of the heterogeneity seen among studies (the β (SE) for continuous and binary overall incidence of cancer for the aforementioned variables respectively are as follow. sex: 0.04 (0.03) and 0.06 (0.07), country of study conduction: 0.003 (0.01) and 0.02 (0.04), cancer type: -0.001 (0.005) and -0.001 (0.01))

### Publication bias

Publication bias was assessed using Egger’s test and was present across the included studies in our analyses. However, after trim fill analysis, only cancer incidence based on continuous measure changed from (HR:1.09 95%CI (1.06, 1.12)) to (HR:1.26 95%CI (1.17, 1.34)), while other pooled HRs remained unchanged indicating that the meta-analysis results were not substantially affected by publication bias.

## Discussion

This systematic review and meta-analysis showed a significant association between childhood and adolescent obesity and malignancies’ overall incidence and mortality in adulthood. Moreover, most cancer incidences, stratified by malignancy type, were directly and significantly associated with obesity under 18 years of age. These findings were in line with other studies as they also reported a significant association between cancer incidence and mortality with obesity in the youth (25, 26). Our findings were in line with other systematic reviews and meta-analyses studying specific malignancies, indicating that obesity in early life is associated with a greater risk of colorectal cancer in adulthood (RR:1.39, 95%CI (1.20, 1.62)) (20), higher risk of ovarian cancer that tends to increase with the increment of BMI (RR:1.19, 95% CI (1.11, 1.28) (21). It should be kept in mind that regardless of the association of obesity with the incidence and mortality of various cancers in adulthood, childhood obesity can persist into adulthood and adulthood obesity itself is an important risk factor for cancer and other morbidities (27). Furthermore, the overall pooled effect size of obesity on all cancer incidences should be interpret cautiously (**Table 1**), and despite the seen significant association, it is advised to consider the effect sizes sub grouped by type of cancer instead (**Figure 2**)

The exact mechanisms by which obesity in the youth affects cancer incidence and mortality in adulthood are not well understood. Some studies suggested the role of chronic inflammation and oxidative DNA damage, hormonal and cytokine dysregulation in obesity as possible underlying causes (25, 26). Indicating the need for studies evaluating possible underlying pathways by which obesity contributes to cancer incidence and mortality.

In a systematic review and meta-analysis similar to this study on those aged under 30, a significant association between obesity and colorectal, endometrial, ovarian, thyroid, and pancreas malignancies were found. Furthermore the aforementioned study found that pre and post-menopausal obesity reduces the risk of breast cancer by 12 and 17 percent respectively (RR:0.88 95%CI (0.81, 0.95) and RR:0.83 95%CI (0.77, 0.89)) (20); our findings were similar to the aforementioned study, however, in our study childhood and adolescent did not have a significant protective effect on breast cancer, probably due to the difference of the targeted population; other studies have also stated that obesity in the youth can be protective for adulthood breast cancer (28, 29). Nonetheless, some other studies argue that obesity can increase the risk and worsen the outcome of breast cancer (30, 31). Since many studies on this subject are retrospective cohorts, possible confounding variables such as predisposing genetic factors, nutritional indices, etc. could be the source of this controversy. Moreover, although height (which is also used in the calculation of BMI) has been associated with the incidence of various cancers in numerous studies (12, 32), all of the included studies adjusted their findings for height and BMI to address its potential confounding properties. Nonetheless, The BMI system has its flaws and inadequacies. As new definitions are offered to represent obesity, the inadequacies and weaknesses within the BMI system become more highlighted. Some types of obesity, such as normal weight obesity (NWO), despite having a normal weight and BMI, are at increased risk of cardiometabolic diseases due to their high-fat proportion (33). Individuals with these types of obesity might also have an increased risk of cancer incidence and mortality as well; however, studies have not addressed this issue yet, indicating the need for cohort studies assessing body fat proportion, metabolic obesity, etc.

Although understanding the pathways by which obesity increases the risk of cancer incidence and mortality is essential in fighting it; regardless of the underlying pathologic pathways, the outcome is quite clear and the fact is that many cohort studies have shown that obesity in the youth increases the risk of malignancies in adulthood (34–36). This is worrisome since the prevalence of obesity is increasing worldwide, especially among the young. Since obesity is a major cardiometabolic risk factor as well, with consideration of its long-lasting effects and contribution to the prevalence of cancers, this increase in the prevalence of obesity needs major attention and intervention (25).

This indicates that more thorough preventive measures must be taken to prevent the upcoming rise in cancer incidence. Moreover, the necessity of proper education cannot be ignored. Since not only education on obesity is needed, but in order to prevent it, we should educate people on how to get rid of obesity; as even now, many minors do suffer from obesity, and governments must take action to prevent and treat obese individuals, as soon as possible to prevent the conditions that arise from obesity.

### Limitations and strengths

In this thorough systematic review and meta-analysis, only cohort studies with documented measurements for BMI were evaluated (37–69), their aggregated data were pooled, and a precise evaluation of the association between obesity indices in the youth and cancers (all-cause and type of cancer incidence and cancer mortality) in adulthood was drawn. However, there were some limitations. As can be seen, these studies were conducted in a handful of countries; hence the findings of this study may differ across other nations and races as many regions and many countries lacked proper cohort studies to evaluate this subject. Moreover, the number of studies on cancer subtypes was relatively low and more studies are needed to determine the association of obesity with various cancer subtypes. Lastly, many of these studies were retrospective cohorts based on registry data, without proper adjustment for confounding variables and such as predisposing genetic factors, nutritional status and socio-economical status.

## Conclusion

This study showed a clear association between childhood and adolescent obesity and cancer incidence and mortality in adulthood. Obesity is associated with an increased prevalence of various types of malignancies and a higher death rate due to cancer in adulthood. With regard to these findings, we must increase our efforts to battle obesity, and interventions in childhood and youth need to be balanced with the need to tackle adult obesity.

## Data availability statement

The original contributions presented in the study are included in the article/**Supplementary Material**. Further inquiries can be directed to the corresponding authors.

## Ethics statement

The Ethic Committee of Alborz university of Medical Sciences (ABZUMS) approved and all methods of the study were carried out by relevant guidelines and regulations.

## Author contributions

NMK conceived the study, participated in study design, data collection (searching the databases, extraction of the data, and assessing the qualities of the manuscripts) and wrote the manuscript. MQ, SA and ES aided in data collection (searching the databases, extraction of the data, and assessing the qualities of the manuscripts), SM, LS, BHZ and AE helped in manuscript preparation. And interpreted the results. All authors contributed to the article and approved the submitted version.
