# Supplementary material for: Association of childhood and adolescence obesity with incidence and mortality of adulthood cancers. A systematic review and meta-analysis
Source: Front Endocrinol (Lausanne). 2023 Jan 19;14:1069164. doi: 10.3389/fendo.2023.1069164 (PMC9892178; doi:10.3389/fendo.2023.1069164)
Supplement: Supplementary file 1 [file Table_1.docx]

| Supplementary Table 1. The search strategy categorized by searched databases. | | | |
| --- | --- | --- | --- |
| **Database** | **Syntax** | **Result** | **NNR** |
| PubMed | ((Obesity* [tiab] AND Pediatric*[tiab]) OR "Obesity in Childhood"[tiab] OR "Childhood Onset Obesity"[tiab] OR (Obesity*[tiab] AND Childhood Onset[tiab]) OR "Child Obesity*"[tiab] OR (Obesity*[tiab] AND Child*[tiab]) OR "Childhood Obesity*"[tiab] OR (Obesity* [tiab] AND Childhood*[tiab]) OR "Adolescent Obesity*"[tiab] OR (Obesity*[tiab] AND Adolescent*[tiab]) OR "Obesity in Adolescence*"[tiab] OR "Infantile Obesity*"[tiab] OR (Obesity* [tiab] AND Infantile[tiab]) OR "Infant Obesity*"[tiab] OR (Obesity*[tiab] AND Infant[tiab]) OR "Childhood Overweight*"[tiab] OR (Overweight* [tiab] AND Childhood*[tiab]) OR "Infant Overweight*"[tiab] OR (Overweight*[tiab] AND Infant*[tiab] ) OR "Adolescent Overweight*"[tiab] OR (Overweight*[tiab] AND Adolescent*[tiab]) OR " Childhood Body-mass index"[tiab] OR "Childhood Body Mass Index"[tiab] OR "Adolescent body mass index"[tiab] OR "Adolescent Body-mass index"[tiab] OR ("Body Mass Index"[tiab] AND child*[tiab]) OR ("Body Mass Index"[tiab] AND adolesce* [tiab]) OR (BMI [tiab] AND adolesce*[tiab]) OR (BMI [tiab] AND Child*[tiab]) OR ("Adipose Tissue"[tiab] AND child [tiab]) OR ("Adipose Tissue"[tiab] AND adolesce* [tiab]) OR ("Body Fat*"[tiab] AND child*[tiab]) OR ("Body Fat"[tiab] AND adolesce*[tiab]) OR (fat [tiab] AND child*[tiab]) OR ("Body Fat"[tiab] AND adolesce*[tiab]) OR (fatne* [tiab] AND child*[tiab]) OR (fatne** [tiab] AND adolesce*[tiab]) OR ("fat mass"[tiab] AND child*[tiab]) OR ("fat mass"[tiab] AND adolesce*[tiab]) OR ("body fat mass*"[tiab] AND child*[tiab]) OR ("body fat mass*"[tiab] AND adolesce**[tiab]) OR ( "Body fat*"[tiab] AND child*[tiab]) OR ( "Body fat*"[tiab] AND adolesce*[tiab])) AND (cancer*[tiab] OR Neoplasia[ti] OR Neoplasias[ti] OR Neoplasm[ti] OR tumor[ti] OR tumour[ti] OR Malignancy[ti] OR Malignancies[ti] OR “Malignant Neoplasms”[ti] OR “Malignant Neoplasm”[ti] OR (Neoplasm[ti] AND Malignant[ti]) OR (Neoplasms[ti] AND Malignant[ti]) OR “Benign Neoplasms”[ti] OR (Neoplasms[ti] AND Benign[ti]) OR “Benign Neoplasm”[ti] OR (Neoplasm[ti] AND Benign[ti]) OR metasta*[ti] OR oncolog*[ti] OR leukemi*[ti] OR lymphoma*[ti] OR myeloma*[ti] OR sarcoma*[ti] OR carcinoma*[ti] OR adenocarcinoma[ti])  AND 1980/01/01:2021/12/20[dp] | 2872 | 16  6/100 |
| Scopus | (TITLE-ABS((Obesity* AND Pediatric*)) OR TITLE-ABS("Obesity in Childhood") OR TITLE-ABS("Childhood Onset Obesity") OR TITLE-ABS((Obesity* AND Childhood Onset)) OR TITLE-ABS("Child Obesity*") OR TITLE-ABS((Obesity*AND Child*)) OR TITLE-ABS("Childhood Obesity*") OR TITLE-ABS((Obesity* AND Childhood*)) OR TITLE-ABS("Adolescent Obesity*") OR TITLE-ABS((Obesity* AND Adolescent*)) OR TITLE-ABS("Obesity in Adolescence*") OR TITLE-ABS("Infantile Obesity*") OR TITLE-ABS((Obesity* AND Infantile)) OR TITLE-ABS( "Infant Obesity*") OR TITLE-ABS((Obesity* AND Infant)) OR TITLE-ABS("Childhood Overweight*") OR TITLE-ABS((Overweight* AND Childhood*)) OR TITLE-ABS("Infant Overweight*") OR TITLE-ABS((Overweight* AND Infant* )) OR TITLE-ABS("Adolescent Overweight*") OR TITLE-ABS((Overweight* AND Adolescent*)) OR TITLE-ABS("Childhood Body-mass index") OR TITLE-ABS("Childhood Body Mass Index") OR TITLE-ABS("Adolescent body mass index") OR TITLE-ABS("Adolescent Body-mass index") OR TITLE-ABS(("Body Mass Index" AND child*)) OR TITLE-ABS(("Body Mass Index" AND adolesce*)) OR TITLE-ABS((BMI AND adolesce*)) OR TITLE-ABS((BMI AND Child*)) OR TITLE-ABS(("Adipose Tissue" AND child )) OR TITLE-ABS(("Adipose Tissue" AND adolesce* )) OR TITLE-ABS(("Body Fat*" AND child*)) OR TITLE-ABS( ("Body Fat" AND adolesce*)) OR TITLE-ABS((fat AND child*)) OR TITLE-ABS( ("Body Fat" AND adolesce*)) OR TITLE-ABS((fatne* AND child*)) OR TITLE-ABS( (fatne** AND adolesce*)) OR TITLE-ABS(("fat mass" AND child*)) OR TITLE-ABS( ("fat mass" AND adolesce*)) OR TITLE-ABS(("body fat mass*" AND child*)) OR TITLE-ABS(("body fat mass*" AND adolesce**)) OR TITLE-ABS(( "Body fat*" AND child*)) OR TITLE-ABS(( "Body fat*" AND adolesce*))) AND (TITLE (cancer*) OR TITLE(Neoplasia) OR TITLE(Neoplasias) OR TITLE(Neoplasm) OR TITLE(tumor) OR TITLE(tumour) OR TITLE(Malignancy) OR TITLE(Malignancies) OR TITLE( “Malignant Neoplasms”) OR TITLE(“Malignant Neoplasm”) OR TITLE((Neoplasm AND Malignant)) OR TITLE((Neoplasms AND Malignant)) OR TITLE(“Benign Neoplasms”) OR TITLE((Neoplasms AND Benign)) OR TITLE(“Benign Neoplasm”) OR TITLE((Neoplasm AND Benign)) OR TITLE(metasta*) OR TITLE(oncolog*) OR TITLE(leukemi*) OR TITLE(lymphoma*) OR TITLE(myeloma*) OR TITLE(sarcoma*) OR TITLE(carcinoma*) OR TITLE(adenocarcinoma))  AND PUBYEAR > 1979 AND PUBYEAR < 2022 | 1827  Conference paper=23 | 10  10/100 |
| WOS | (TS= ((Obesity* AND Pediatric*)) OR TS=("Obesity in Childhood") OR TS= ("Childhood Onset Obesity") OR TS= ((Obesity* AND Childhood Onset)) OR TS= ("Child Obesity*") OR TS=((Obesity*AND Child*)) OR TS=("Childhood Obesity*") OR TS= ((Obesity* AND Childhood*)) OR TS=("Adolescent Obesity*") OR TS= ((Obesity* AND Adolescent*)) OR TS=("Obesity in Adolescence*") OR TS=("Infantile Obesity*") OR TS=((Obesity* AND Infantile)) OR TS=( "Infant Obesity*") OR TS=((Obesity* AND Infant)) OR TS=("Childhood Overweight*") OR TS=((Overweight* AND Childhood*)) OR TS=("Infant Overweight*") OR TS=((Overweight* AND Infant* )) OR TS= ("Adolescent Overweight*") OR TS=((Overweight* AND Adolescent*)) OR TS= ("Childhood Body-mass index") OR TS=("Childhood Body Mass Index") OR TS= ("Adolescent body mass index") OR TS=("Adolescent Body-mass index") OR TS= (("Body Mass Index" AND child*)) OR TS=(("Body Mass Index" AND adolesce*)) OR TS=((BMI AND adolesce*)) OR TS=((BMI AND Child*)) OR TS=(("Adipose Tissue" AND child )) OR TS=(("Adipose Tissue" AND adolesce* )) OR TS= (("Body Fat*" AND child*)) OR TS=( ("Body Fat" AND adolesce*)) OR TS=((fat AND child*)) OR TS=( ("Body Fat" AND adolesce*)) OR TS=((fatne* AND child*)) OR TS=((fatne** AND adolesce*)) OR TS=(("fat mass" AND child*)) OR TS=(("fat mass" AND adolesce*)) OR TS=(("body fat mass*" AND child*)) OR TS=(("body fat mass*" AND adolesce**)) OR TS=(( "Body fat*" AND child*)) OR TS=(( "Body fat*" AND adolesce*))) AND (TS= (cancer*) OR TI=(Neoplasia) OR TI=(Neoplasias) OR TI=(Neoplasm) OR TI=(tumor) OR TI=(tumour) OR TI=(Malignancy) OR TI=(Malignancies) OR TI=( “Malignant Neoplasms”) OR TI=(“Malignant Neoplasm”) OR TI=((Neoplasm AND Malignant)) OR TI=((Neoplasms AND Malignant)) OR TI=(“Benign Neoplasms”) OR TI= ((Neoplasms AND Benign)) OR TI=(“Benign Neoplasm”) OR TI=((Neoplasm AND Benign)) OR TI=(metasta*) OR TI=(oncolog*) OR TI=(leukemi*) OR TI= (lymphoma*) OR TI=(myeloma*) OR TI=(sarcoma*) OR TI=(carcinoma*) OR TI= (adenocarcinoma))  AND PY= (1980-2021) | 4602  Proceeding paper=181  Meeting abstract=90 | 20  5/100 |

| Supplementary Table 2. General characteristics of the included studies | | | | | | | | |
| --- | --- | --- | --- | --- | --- | --- | --- | --- |
| First Author (year) | Country | Measure | Sex | Type of Cancer or Cancer Mortality | Cancer Subgroup | Age range (year) | Sample size | Cancer number |
| Aarestrup 2014 (PRE PSA) (37) | Denmark | BMI (1 unit increase BMI Z score) | Male | Prostate | (-) | 7 | 95049 | 189 |
|  |  |  |  |  |  | 8 | 96790 | 197 |
|  |  |  |  |  |  | 9 | 96727 | 197 |
|  |  |  |  |  |  | 10 | 96621 | 197 |
|  |  |  |  |  |  | 11 | 96547 | 198 |
|  |  |  |  |  |  | 12 | 96095 | 194 |
|  |  |  |  |  |  | 13 | 95030 | 186 |
| Aarestrup 2014 (POST PSA) (37) |  |  |  |  |  | 7 | 107734 | 1522 |
|  |  |  |  |  |  | 8 | 109796 | 1540 |
|  |  |  |  |  |  | 9 | 109584 | 1536 |
|  |  |  |  |  |  | 10 | 109473 | 1534 |
|  |  |  |  |  |  | 11 | 109531 | 1538 |
|  |  |  |  |  |  | 12 | 109103 | 1534 |
|  |  |  |  |  |  | 13 | 108136 | 1525 |
| Aarestrup 2017 (38) | Denmark | BMI (1 unit increase BMI Z score) | Female | Endometrial | Estrogen Dependent | 7 | 994287 | 920 |
|  |  |  |  |  | Adenocarcinoma |  |  | 659 |
|  |  |  |  |  | Total |  |  | 1020 |
|  |  | BMI (0.1 unit increase BMI Z score) |  |  | Total | 6.25-7.99 |  |  |
|  |  |  |  |  |  | 8-10.99 |  |  |
|  |  |  |  |  |  | 11 to 14 |  |  |
|  |  |  |  |  |  | total |  |  |
|  |  |  |  |  | Estrogen Dependent | 6.25-7.99 |  | 920 |
|  |  |  |  |  |  | 8-10.99 |  |  |
|  |  |  |  |  |  | 11 to 14 |  |  |
|  |  |  |  |  |  | total |  |  |
|  |  |  |  |  | Adenocarcinoma | 6.25-7.99 |  | 659 |
|  |  |  |  |  |  | 8-10.99 |  |  |
|  |  |  |  |  |  | 11 to 14 |  |  |
|  |  |  |  |  |  | total |  |  |
| Aarestrup 2016 (39) | Denmark | BMI (1 unit increase BMI Z score) | Female | Endometrial | Non Estrogen Dependent | 7 | 145406 | 84 |
|  |  |  |  |  |  | 8 | 147098 | 86 |
|  |  |  |  |  |  | 9 | 142094 | 85 |
|  |  |  |  |  |  | 10 | 138587 | 87 |
|  |  |  |  |  |  | 11 | 137678 | 86 |
|  |  |  |  |  |  | 12 | 136614 | 86 |
|  |  |  |  |  |  | 13 | 134707 | 88 |
|  |  |  |  |  | Adenocarcinoma | 7 | 131120 | 501 |
|  |  |  |  |  |  | 8 | 132493 | 512 |
|  |  |  |  |  |  | 9 | 127482 | 512 |
|  |  |  |  |  |  | 10 | 123940 | 508 |
|  |  |  |  |  |  | 11 | 123007 | 510 |
|  |  |  |  |  |  | 12 | 121950 | 510 |
|  |  |  |  |  |  | 13 | 120133 | 498 |
| Aarestrup 2019 (9) | Denmark | overweight/normal | Female | Ovaries | Total | 7 | 9455/136366 | 61/923 |
|  |  |  |  |  |  | 8 | 10137/137382 | 62/932 |
|  |  |  |  |  |  | 9 | 10373/132145 | 71/931 |
|  |  |  |  |  |  | 10 | 10024/128979 | 67/917 |
|  |  |  |  |  |  | 11 | 9501/128602 | 63/930 |
|  |  |  |  |  |  | 12 | 9490/127551 | 65/922 |
|  |  |  |  |  |  | 13 | 9764/125366 | 71/908 |
|  |  |  |  |  | Serous | 7 | (-) | 23/510 |
|  |  |  |  |  |  | 8 |  | 21/521 |
|  |  |  |  |  |  | 9 |  | 30/505 |
|  |  |  |  |  |  | 10 |  | 31/509 |
|  |  |  |  |  |  | 11 |  | 28/512 |
|  |  |  |  |  |  | 12 |  | 30/510 |
|  |  |  |  |  |  | 13 |  | 28/512 |
|  |  |  |  |  | Mucinous | 7 |  | 12/95 |
|  |  |  |  |  |  | 8 |  | 11/94 |
|  |  |  |  |  |  | 9 |  | 11/94 |
|  |  |  |  |  |  | 10 |  | 11/92 |
|  |  |  |  |  |  | 11 |  | 10/96 |
|  |  |  |  |  |  | 12 |  | 9/96 |
|  |  |  |  |  |  | 13 |  | 11/93 |
|  |  |  |  |  | Endometrioid | 7 |  | 8/91 |
|  |  |  |  |  |  | 8 |  | 9/90 |
|  |  |  |  |  |  | 9 |  | 11/86 |
|  |  |  |  |  |  | 10 |  | 9/87 |
|  |  |  |  |  |  | 11 |  | 10/87 |
|  |  |  |  |  |  | 12 |  | 10/87 |
|  |  |  |  |  |  | 13 |  | 10/86 |
|  |  |  |  |  | Clear Cell | 7 |  | 5/32 |
|  |  |  |  |  |  | 8 |  | 6/31 |
|  |  |  |  |  |  | 9 |  | 7/31 |
|  |  |  |  |  |  | 10 |  | 6/32 |
|  |  |  |  |  |  | 11 |  | 4/34 |
|  |  |  |  |  |  | 12 |  | 6/33 |
|  |  |  |  |  |  | 13 |  | 6/32 |
|  |  |  |  |  | Other | 7 |  | 13/195 |
|  |  |  |  |  |  | 8 |  | 15/196 |
|  |  |  |  |  |  | 9 |  | 12/197 |
|  |  |  |  |  |  | 10 |  | 10/197 |
|  |  |  |  |  |  | 11 |  | 11/201 |
|  |  |  |  |  |  | 12 |  | 10/196 |
|  |  |  |  |  |  | 13 |  | 16/185 |
| Ahlgren 2006 (11) | Denmark | BMI (1 unit increase BMI ) | Female | Breast | (-) | under 8 | 141393 | 3340 |
|  |  |  |  |  |  | 8 to 14 |  |  |
| Anderson 2014 (30) | Denmark | BMI (1 unit increase BMI Z score) | Female | Breast | (-) | 7 | 13572 | 716 |
|  |  |  |  |  |  | 8 |  |  |
|  |  |  |  |  |  | 9 |  |  |
|  |  |  |  |  |  | 10 |  |  |
|  |  |  |  |  |  | 11 |  |  |
|  |  |  |  |  |  | 12 |  |  |
|  |  |  |  |  |  | 13 |  |  |
| T. L. Berentzen 2014 (40) | Denmark | BMI (1 unit increase BMI Z score) | Both | Liver | Primary Liver Cancer | 7 | 263532 | 240 |
|  |  |  |  |  |  | 8 | 267722 | 244 |
|  |  |  |  |  |  | 9 | 262629 | 248 |
|  |  |  |  |  |  | 10 | 257888 | 250 |
|  |  |  |  |  |  | 11 | 256222 | 250 |
|  |  |  |  |  |  | 12 | 253848 | 249 |
|  |  |  |  |  |  | 13 | 250300 | 243 |
|  |  |  |  |  | HCC | 7 | 263532 | 143 |
|  |  |  |  |  |  | 8 | 267722 | 146 |
|  |  |  |  |  |  | 9 | 262629 | 146 |
|  |  |  |  |  |  | 10 | 257888 | 149 |
|  |  |  |  |  |  | 11 | 256222 | 151 |
|  |  |  |  |  |  | 12 | 253848 | 151 |
|  |  |  |  |  |  | 13 | 250300 | 146 |
| T. Bjørge 2008 (31) | Norway | BMI quartile (75-84) | Male | Cancer Mortality | Colon | 14-19 | 307375 | 7 |
|  |  |  |  |  | Respiratory |  |  | 16 |
|  |  |  |  |  | Total |  |  | 101 |
|  |  |  |  |  | Hematologic/Lymphatic |  |  | 15 |
|  |  |  | Female |  | Colon |  | 443257 | 16 |
|  |  |  |  |  | Respiratory |  |  | 16 |
|  |  |  |  |  | Hematologic/Lymphatic |  |  | 21 |
|  |  |  |  |  | Breast |  |  | 55 |
|  |  |  |  |  | Total |  |  | 193 |
|  |  |  |  |  | Cervix |  |  | 12 |
|  |  |  |  |  | Ovary` |  |  | 16 |
|  |  | BMI quartile (above 84) | Male | Cancer Mortality | Colon |  | 207816 | 10 |
|  |  |  |  |  | Respiratory |  |  | 13 |
|  |  |  |  |  | Total |  |  | 77 |
|  |  |  |  |  | Hematologic/Lymphatic |  |  | 13 |
|  |  |  | Female |  | Colon |  | 312180 | 16 |
|  |  |  |  |  | Respiratory |  |  | 18 |
|  |  |  |  |  | Hematologic/Lymphatic |  |  | 14 |
|  |  |  |  |  | Breast |  |  | 31 |
|  |  |  |  |  | Cervix |  |  | 17 |
|  |  |  |  |  | Total |  |  | 166 |
|  |  |  |  |  | Ovary` |  |  | 7 |
| T. Bjørge 2004 (41) | Norway | BMI quartile (75-84) | Male | RCC | (-) | 14-19 | male 115267, female 111954 | 13 |
|  |  |  | Female |  |  |  |  | 7 |
|  |  | BMI quartile (above 84) | Male |  |  |  |  | 14 |
|  |  |  | Female |  |  |  |  | 5 |
| T. Bjørge 2006 (42) | Norway | BMI quartile (75-84) | Male | Testicular Cancer | Seminoma | 14-19 | 288236 | (-) |
|  |  |  |  |  | Non-Seminoma |  |  |  |
|  |  |  |  |  | Total |  |  | 41 |
|  |  | BMI quartile (above 84) |  |  | Seminoma |  | 194911 | (-) |
|  |  |  |  |  | Non-Seminoma |  |  |  |
|  |  |  |  |  | Total |  |  | 23 |
| Celind 2020 (7) | Sweden | BMI (1 unit increase BMI SD) | Both | Hematologic | (-) | 8 | 37669 | 459 |
| Celind 2019 (43) | Sweden | BMI (1 unit increase BMI SD) | Both | GI | Rectum | 8 | 37663 | 257 colon and 157 rectum |
|  |  |  |  |  | Colon |  |  |  |
|  |  | overweight/normal |  |  | Rectum |  |  |  |
|  |  |  |  |  | Colon |  |  |  |
| Cook 2015 (44) | Denmark | BMI (1 unit increase BMI Z score) | Male | GI | Esophagus | 7 | 121037 | 207 |
|  |  |  |  |  |  | 8 | 123359 | 207 |
|  |  |  |  |  |  | 9 | 123157 | 206 |
|  |  |  |  |  |  | 10 | 123063 | 206 |
|  |  |  |  |  |  | 11 | 123103 | 208 |
|  |  |  |  |  |  | 12 | 122431 | 207 |
|  |  |  |  |  |  | 13 | 120332 | 206 |
|  |  |  | Female |  |  | 7 | 119398 | 34 |
|  |  |  |  |  |  | 8 | 121781 | 34 |
|  |  |  |  |  |  | 9 | 121809 | 35 |
|  |  |  |  |  |  | 10 | 121971 | 35 |
|  |  |  |  |  |  | 11 | 122190 | 37 |
|  |  |  |  |  |  | 12 | 121910 | 37 |
|  |  |  |  |  |  | 13 | 120581 | 36 |
|  |  |  | Both |  |  | 7 | 240435 | 241 |
|  |  |  |  |  |  | 8 | 245140 | 241 |
|  |  |  |  |  |  | 9 | 244966 | 241 |
|  |  |  |  |  |  | 10 | 245034 | 241 |
|  |  |  |  |  |  | 11 | 245293 | 245 |
|  |  |  |  |  |  | 12 | 244341 | 244 |
|  |  |  |  |  |  | 13 | 240913 | 241 |
| De Stavola 2004 (28) | UK | BMI (1 unit increase BMI SD) | Female | Breast | (-) | 2 | 1705 | 50 |
|  |  |  |  |  |  | 4 | 1903 | 55 |
|  |  |  |  |  |  | 7 | 1853 | 52 |
|  |  |  |  |  |  | 11 | 1836 | 51 |
|  |  |  |  |  |  | 15 | 1664 | 43 |
| Engeland 2003 (45) | Norway | BMI quartile (75-84) | Female | Ovaries | (-) | 14 to 19 | 111886 | 38 |
|  |  | BMI quartile (above 84) |  |  |  |  |  | 29 |
| Farfel 2014 (12) | Israel | BMI 4th quintile | Male | Thyroid | (-) | 16 to 19 | 1624310 | 437 male, 323 female |
|  |  |  | Female |  |  |  |  |  |
|  |  | BMI 5th quintile | Male |  |  |  |  |  |
|  |  |  | Female |  |  |  |  |  |
| Furer 2020 (34) | Israel | BMI percentile | Male | Various | Total | 16 to 19 | 320517 | 6518 |
|  |  |  | Female |  |  |  | 256814 | 8723 |
|  |  |  | Male |  |  |  | 116233 | 2354 |
|  |  |  | Female |  |  |  | 99853 | 3173 |
|  |  |  | Male |  |  |  | 108820 | 2054 |
|  |  |  | Female |  |  |  | 86152 | 2421 |
|  |  |  | Male |  |  |  | 59520 | 861 |
|  |  |  | Female |  |  |  | 26541 | 451 |
|  |  |  | Both (First Cohort) |  |  |  | 195909 | 2326 |
|  |  |  | Both (Second Cohort) |  |  |  | 286975 | 3773 |
|  |  |  | Both (First Cohort) |  |  |  | 46917 | 609 |
|  |  |  | Both (Second Cohort) |  |  |  | 90990 | 1267 |
|  |  |  | Both (First Cohort) |  | Brain |  | 2458170 | 515 |
|  |  |  | Both (Second Cohort) |  | Brain |  |  | 770 |
|  |  |  | Both (First Cohort) |  | Thyroid |  |  | 428 |
|  |  |  | Both (Second Cohort) |  | Thyroid |  |  | 1416 |
|  |  |  | Both (First Cohort) |  | Non Hodgkin Lymphoma |  |  | 570 |
|  |  |  | Both (Second Cohort) |  | Non-Hodgkin Lymphoma |  |  | 1113 |
|  |  |  | Both (First Cohort) |  | Colorectal Cancer |  |  | 327 |
|  |  |  | Both (Second Cohort) |  | Colorectal Cancer |  |  | 482 |
|  |  |  | Both (First Cohort) |  | Oral Cavity Cancer |  |  | 327 |
|  |  |  | Both (Second Cohort) |  | Oral Cavity Cancer |  |  | 203 |
|  |  |  | Both (First Cohort) |  | Stomach Cancer |  |  | 106 |
|  |  |  | Both (Second Cohort) |  | Stomach Cancer |  |  | 120 |
|  |  |  | Both (First Cohort) |  | Hodgkin Lymphoma |  |  | 427 |
|  |  |  | Both (Second Cohort) |  | Hodgkin Lymphoma |  |  | 961 |
|  |  |  | Both (First Cohort) |  | Leukemia |  |  | 243 |
|  |  |  | Both (Second Cohort) |  | Leukemia |  |  | 347 |
|  |  |  | Both (First Cohort) |  | Cervical Cancer |  |  | 710 |
|  |  |  | Both (Second Cohort) |  | Cervical Cancer |  |  | 3051 |
|  |  |  | Female (First Cohort) |  | Ovarian Cancer |  |  | 116 |
|  |  |  | Female (Second Cohort) |  | Ovarian Cancer |  |  | 161 |
|  |  |  | Male (First Cohort) |  | Testis Cancer |  |  | 363 |
|  |  |  | Male (Second Cohort) |  | Testis Cancer |  |  | 880 |
|  |  |  | Female (First Cohort) |  | Breast Cancer |  |  | 1288 |
|  |  |  | Female (Second Cohort) |  | Breast Cancer |  |  | 2610 |
|  |  |  | Both (First Cohort) |  | Melanoma |  |  | 1198 |
|  |  |  | Both (Second Cohort) |  | Melanoma |  |  | 1786 |
|  |  |  | Male | Cancer Survival | Total |  | 69207 | 1978 |
|  |  |  | Female |  |  |  | 103082 | 1575 |
|  |  |  | Male |  |  |  | 24747 | 726 |
|  |  |  | Female |  |  |  | 37876 | 611 |
|  |  |  | Male |  |  |  | 21209 | 658 |
|  |  |  | Female |  |  |  | 27383 | 473 |
|  |  |  | Male |  |  |  | 8212 | 310 |
|  |  |  | Female |  |  |  | 4534 | 110 |
| Hagström 2018 (46) | Sweden | BMI ( overweight/normal) | Male | HCC | (-) | 17 to 19 | 1220261 | 47 |
|  |  |  |  |  |  |  |  | 25 |
|  |  |  |  |  |  |  |  | 10 |
| Jeffreys 2004 (47) | UK | BMI (1 unit increase BMI SD) | Both | Colorectal | (-) | 2 to 14 | 2309 | 38 |
|  |  |  | Male | Prostate | (-) |  | 1138 | 19 |
|  |  |  | Female | Breast | (-) |  | 1132 | 58 |
|  |  |  | Both | Lung | (-) |  | 2263 | 84 |
| Jensen 2018 (48) | Denmark | BMI ( overweight/normal) | Male | Colon | (-) | 7 to 26 (7 to 19) | 704 /54273 | 17/614 |
| Jensen 2017 (49) | Denmark | BMI (1 unit increase BMI Z score) | Male | Non-Sigmoid Colon | (-) | 7 | 122203 | 707 |
|  |  |  |  |  |  | 10 | 124225 | 715 |
|  |  |  |  |  |  | 13 | 120506 | 692 |
|  |  |  |  | Sigmoid Colon | (-) | 7 | 122203 | 602 |
|  |  |  |  |  |  | 10 | 124225 | 603 |
|  |  |  |  |  |  | 13 | 120506 | 597 |
|  |  |  | Female | Non-Sigmoid Colon | (-) | 7 | 120736 | 690 |
|  |  |  |  |  |  | 10 | 123301 | 697 |
|  |  |  |  |  |  | 13 | 120878 | 694 |
|  |  |  |  | Sigmoid Colon | (-) | 7 | 120736 | 488 |
|  |  |  |  |  |  | 10 | 123301 | 502 |
|  |  |  |  |  |  | 13 | 120878 | 503 |
|  |  |  | Both | Non-Sigmoid Colon | (-) | 7 | 242939 | 1397 |
|  |  |  |  |  |  | 10 | 247526 | 1412 |
|  |  |  |  |  |  | 13 | 241384 | 1386 |
|  |  |  |  | Sigmoid Colon | (-) | 7 | 242939 | 1090 |
|  |  |  |  |  |  | 10 | 247526 | 1105 |
|  |  |  |  |  |  | 13 | 241384 | 1100 |
| Jensen 2020 (8) | Denmark | BMI ( overweight/normal) | Both | Renal Cell Carcinoma | (-) | 7 | 13739/269077 | 32/918 |
|  |  |  |  |  |  | 10 | 15723/256472 | 46/912 |
|  |  |  |  |  |  | 13 | 16380/247025 | 62/890 |
| Kantor 2016 (50) | Sweden | BMI ( lower overweight/normal) | Males | Colorectal Cancer | (-) | 16 to 20 | 11866/193828 | 48/698 |
|  |  | BMI ( upper overweight/normal) |  |  |  |  | 3694/193828 | 27/698 |
|  |  | BMI ( obese/normal) |  |  |  |  | 2364/193828 | 20/698 |
| Katz 2018 (51) | Israel | BMI percentile (85th( overweight and obese)/normal) | Both | Gep-Net | (-) | 16 to 19 | 2298130 | 221 |
| Keinan 2020 (52) | Israel | BMI ( overweight/normal) | Female | Cervix | (-) | 17 | 969123 | 859 |
|  |  | BMI ( obese /normal) |  |  |  |  |  |  |
| Keinan 2016 (29) | Israel | BMI ( overweight/normal) | Female | Breast | (-) | 16-19 | 103261/951480 | 834/9660 |
|  |  | BMI ( obese /normal) |  |  |  |  | 26739/951480 | 89ob/9660nw |
| Keinan 2018 (53) | Israel | BMI ( overweight/normal) | Male | Breast | Male Breast Cancer | 17 | 144076/1014245 | 16/66 |
|  |  | BMI ( obese /normal) |  |  |  |  | 39743/1014245 | 6/66 |
| Kitahara 2014 (54) | Denmark | BMI (1 unit increase BMI SDs) | Male | Thyroid | (-) | 7 | 151914 | 61 |
|  |  |  |  |  |  | 8 | 153750 | 63 |
|  |  |  |  |  |  | 9 | 147881 | 61 |
|  |  |  |  |  |  | 10 | 143839 | 61 |
|  |  |  |  |  |  | 11 | 142687 | 61 |
|  |  |  |  |  |  | 12 | 141218 | 60 |
|  |  |  |  |  |  | 13 | 138616 | 58 |
|  |  |  | Female |  |  | 7 | 151891 | 163 |
|  |  |  |  |  |  | 8 | 153737 | 166 |
|  |  |  |  |  |  | 9 | 147871 | 162 |
|  |  |  |  |  |  | 10 | 143832 | 162 |
|  |  |  |  |  |  | 11 | 142680 | 164 |
|  |  |  |  |  |  | 12 | 141214 | 162 |
|  |  |  |  |  |  | 13 | 138610 | 158 |
|  |  |  | Both | Thyroid | Papillary | 7 | 299897 | 137 |
|  |  |  |  |  |  | 8 | 303522 | 140 |
|  |  |  |  |  |  | 9 | 292658 | 135 |
|  |  |  |  |  |  | 10 | 285104 | 135 |
|  |  |  |  |  |  | 11 | 283061 | 137 |
|  |  |  |  |  |  | 12 | 280539 | 135 |
|  |  |  |  |  |  | 13 | 276037 | 132 |
|  |  |  |  |  | Follicular | 7 | 299897 | 56 |
|  |  |  |  |  |  | 8 | 303522 | 58 |
|  |  |  |  |  |  | 9 | 292658 | 57 |
|  |  |  |  |  |  | 10 | 285104 | 56 |
|  |  |  |  |  |  | 11 | 283061 | 56 |
|  |  |  |  |  |  | 12 | 280539 | 56 |
|  |  |  |  |  |  | 13 | 276037 | 54 |
| Kitahara 2014 (55) | Denmark | BMI (1 unit increase BMI SDs) | Male | Glioblastoma | (-) | 7 | 151667 | 206 |
|  |  |  |  |  |  | 8 | 153501 | 207 |
|  |  |  |  |  |  | 9 | 147644 | 206 |
|  |  |  |  |  |  | 10 | 143601 | 205 |
|  |  |  |  |  |  | 11 | 142453 | 211 |
|  |  |  |  |  |  | 12 | 140986 | 210 |
|  |  |  |  |  |  | 13 | 138363 | 207 |
|  |  |  | Female |  |  | 7 | 147807 | 125 |
|  |  |  |  |  |  | 8 | 149570 | 130 |
|  |  |  |  |  |  | 9 | 144575 | 131 |
|  |  |  |  |  |  | 10 | 141064 | 133 |
|  |  |  |  |  |  | 11 | 140175 | 130 |
|  |  |  |  |  |  | 12 | 139111 | 131 |
|  |  |  |  |  |  | 13 | 137195 | 131 |
| Landberg 2019 (10) | Sweden | BMI ( overweight/normal) | Male | RCC | (-) | 16 to 20 | 15507/193166 | 27/198 |
|  |  | BMI ( obese /normal) |  |  |  |  | 2322/193166 | 7/198 |
|  |  | BMI (1 unit BMI increase) |  |  |  |  | 238788 | 266 |
| Leiba 2017 (56) | Israel | BMI ( overweight/normal) | Both | Hematologic | Myeloproliferative Malignancies | 16 to 19 | 209431/1982609 | 433 |
|  |  | BMI ( obese /normal) |  |  |  |  | 94664/1982609 |  |
| Leiba 2013 (57) | Israel | BMI (2.5 unit BMI increase) | Male | RCC | (-) | 16 to 20 | 197755/778758 | 61/173 |
|  |  |  |  |  |  |  | 76802/778758 | 19/173 |
|  |  |  |  |  |  |  | 57520/778758 | 21/173 |
| Leiba 2012 (58) | Israel | BMI percentile | Both | Urothelial Cancer | (-) | 16 to 19 | 2104238/17472397 | 89/572 |
| Leiba 2016 (35) | Israel | BMI percentile | Both | Non Hodgkin Lymphoma | 9 | 16 to 19 | 2352988 | 4021 |
|  |  | BMI (5 unit BMI increase) |  |  |  |  |  |  |
| Levi 2012 (59) | Israel | BMI percentile | Both | Pancreas | (-) | 16 to 19 | 74991/645936 | 18/80 |
|  |  | BMI (above 1zscore/1zscore) |  |  |  |  | 96941/562501 | 24/65 |
| Levi 2011 (60) | Israel | BMI percentile | Male | GI | Colon | 16 to 19 | 138471/971393 | 445 |
|  |  |  |  |  | Rectum |  |  | 193 |
|  |  |  |  |  | Non-Mucinous Colorectal |  |  | 537 |
|  |  |  |  |  | Non-Mucinous Colon |  |  | (-) |
|  |  |  |  |  | Mucinous Colorectal |  |  | 101 |
| Levi 2017 (61) | Israel | BMI ( overweight/normal) | Male | GI | Colon | 16 to 19 | 78416/881845 | 133/1113 |
|  |  | BMI ( obese /normal) |  |  |  |  | 37864/881845 | 46/1113 |
|  |  | BMI ( overweight/normal) | Female |  |  |  | 62051/597967 | 85/629 |
|  |  | BMI ( obese /normal) |  |  |  |  | 16360/597967 | 14/629 |
|  |  | BMI ( overweight/normal) | Both |  |  |  | 140467/1479812 | 218/1742 |
|  |  | BMI ( obese /normal) |  |  |  |  | 54224/1479812 | 60/1742 |
|  |  | BMI ( overweight/normal) | Male |  | Rectum |  | 78416/881845 | 38/449 |
|  |  | BMI ( obese /normal) |  |  |  |  | 37864/881845 | 21/449 |
|  |  | BMI ( overweight/normal) | Female |  |  |  | 62051/597967 | 15/201 |
|  |  | BMI ( obese /normal) |  |  |  |  | 16360/597967 | 6/201 |
|  |  | BMI ( overweight/normal) | Both |  |  |  | 140467/1479812 | 53/650 |
|  |  | BMI ( obese /normal) |  |  |  |  | 54224/1479812 | 27/650 |
| Levi 2013 (36) | Israel | BMI percentile | Male | GI | EAC | 16 to 19 | 132572/955572 | 4/24 |
|  |  |  |  |  | GEJAC |  |  | 8/16 |
|  |  |  |  |  | EAC And GEJAC |  |  | 10/42 |
|  |  |  |  |  | NCGC |  |  | 15/115 |
|  |  |  |  |  | Intestinal |  |  | 10/67 |
|  |  |  |  |  | Mucinous |  |  | 5/48 |
| Levi 2018 (62) | Israel | BMI ( overweight/normal) | Both | NCGC | (-) | 16 to 19 | 140467/1479812 | 37/425 |
|  |  | BMI ( obese /normal) |  |  |  |  | 54224/1479812 | 19/425 |
|  |  | BMI ( overweight/normal) | Male |  |  |  | 78416/881845 | 31/306 |
|  |  | BMI ( obese /normal) |  |  |  |  | 37864/881845 | 14/306 |
|  |  | BMI ( overweight/normal) | Female |  |  |  | 62051/597967 | 6/119 |
|  |  | BMI ( obese /normal) |  |  |  |  | 16360/597967 | 5/119 |
| Meyle 2017 (63) | Denmark | BMI (1 unit increase BMI Z score) | Both | Melanoma | (-) | 7 | 299891 | 2194 |
|  |  |  |  |  |  | 8 | 303519 | 2227 |
|  |  |  |  |  |  | 9 | 292652 | 2207 |
|  |  |  |  |  |  | 10 | 285093 | 2176 |
|  |  |  |  |  |  | 11 | 283049 | 2178 |
|  |  |  |  |  |  | 12 | 280523 | 2164 |
|  |  |  |  |  |  | 13 | 276020 | 2144 |
| Nogueira 2017 (64) | Denmark | BMI z score 1.5 and above | Both | Pancreatic Cancer | (-) | never | 217721 | 789 |
|  |  |  |  |  |  | 7 | 3829 | 17 |
|  |  |  |  |  |  | 10 | 2131 | 11 |
|  |  |  |  |  |  | 13 | 4243 | 21 |
|  |  |  |  |  |  | 7 and 10 | 1915 | 6 |
|  |  |  |  |  |  | 7 and 13 | 830 | 3 |
|  |  |  |  |  |  | 10 and 13 | 2996 | 13 |
|  |  |  |  |  |  | 7, 10 and 13 | 5204 | 24 |
|  |  |  |  |  |  | never | 50105 | 224 |
|  |  |  |  |  |  | 7 | 753 | 3 |
|  |  |  |  |  |  | 10 | 369 | 2 |
|  |  |  |  |  |  | 13 | 703 | 4 |
|  |  |  |  |  |  | 7 and 10 | 255 | 1 |
|  |  |  |  |  |  | 7 and 13 | 131 | 0 |
|  |  |  |  |  |  | 10 and 13 | 481 | 0 |
|  |  |  |  |  |  | 7, 10 and 13 | 566 | 1 |
|  |  |  |  |  |  | 7 | 275525 | 922 |
|  |  |  |  |  |  | 8 | 279796 | 928 |
|  |  |  |  |  |  | 9 | 273388 | 930 |
|  |  |  |  |  |  | 10 | 267867 | 930 |
|  |  |  |  |  |  | 11 | 266091 | 922 |
|  |  |  |  |  |  | 12 | 263328 | 929 |
|  |  |  |  |  |  | 13 | 259342 | 927 |
|  |  |  |  |  |  | 7 | 58475 | 264 |
|  |  |  |  |  |  | 8 | 59813 | 270 |
|  |  |  |  |  |  | 9 | 59989 | 275 |
|  |  |  |  |  |  | 10 | 60015 | 275 |
|  |  |  |  |  |  | 11 | 60128 | 278 |
|  |  |  |  |  |  | 12 | 59898 | 271 |
|  |  |  |  |  |  | 13 | 59043 | 264 |
| Nuotio 2021 (65) | ? | BMI (1 unit increase SD) | Both | Cancer Mortality | (-) | 3 to 19 | 7405 | 113 |
|  | Finland |  |  |  |  |  | 3572 | 19 |
|  | Australia |  |  |  |  |  | 3200 | 19 |
|  | USA |  |  |  |  |  | 13832 | 312 |
| Petrick 2019 (66) | Denmark | BMI ( obese /normal) | Both | GI | EAC | 7 | 1817/60773 | 11/130 |
|  |  |  |  |  | GCA | 7 |  | 5/120 |
|  |  |  |  |  | Total | 7 |  | 16/250 |
|  |  |  |  |  | EAC | 13 | 3309/60013 | 13/131 |
|  |  |  |  |  | GCA | 13 |  | 10/117 |
|  |  |  |  |  | Total | 13 |  | 23/248 |
|  |  |  |  |  | EAC | 18 | 5300/59395 | 22/124 |
|  |  |  |  |  | GCA | 18 |  | 9/120 |
|  |  |  |  |  | Total | 18 |  | 31/244 |
|  |  |  |  |  | Total | never over weight | 54289 | 218 |
|  |  |  |  |  |  | began at age 7 | 531 | 0 |
|  |  |  |  |  |  | began at age 13 | 10008 | 8 |
|  |  |  |  |  |  | began at age 18 | 2996 | 14 |
|  |  |  |  |  |  | between 7 to 13 | 370 | 0 |
|  |  |  |  |  |  | between 7 to 18 | 176 | 0 |
|  |  |  |  |  |  | between 13 to 18 | 1142 | 6 |
|  |  |  |  |  |  | persistently obese | 705 | 8 |
| Shamriz 2017 (67) | Israel | BMI (continuous) | Both | Acute Myeloid Leukemia | (-) | 16 to 19 | 2310922 | 568 |
| Sørensen 2020 (68) | Denmark | BMI (1 unit increase BMI Z score) | Both | Bladder Cancer | (-) | 7 | 295275 | 1145 |
|  |  |  |  |  |  | 8 | 298756 |  |
|  |  |  |  |  |  | 9 | 287927 |  |
|  |  |  |  |  |  | 10 | 280339 |  |
|  |  |  |  |  |  | 11 | 278341 |  |
|  |  |  |  |  |  | 12 | 275821 |  |
|  |  |  |  |  |  | 13 | 271322 |  |
| Zohar 2019 (69) | Israel | BMI ( overweight/normal) | Both | Pancreatic Cancer | (-) | 16 to 19 | 140467/1479812 | 56/420 |
|  |  | BMI ( obese /normal) |  |  |  |  | 54224/1479812 | 36/420 |
|  |  | BMI ( overweight/normal) | Male |  |  |  | 78416/881845 | 45/315 |
|  |  | BMI ( obese /normal) |  |  |  |  | 37864/881845 | 30/315 |
|  |  | BMI ( overweight/normal) | Female |  |  |  | 62051/597967 | 11/105 |
|  |  | BMI ( obese /normal) |  |  |  |  | 16360/597967 | 6o105 |
| HCC: hepatocellular carcinoma, RCC: renal cell carcinoma, GI: gastrointestinal, Gep-Net: Gastroenteropancreatic Neuroendocrine Tumors, EAC: Esophageal Adenocarcinoma, GEJAC: Gastroesophageal Junction Adenoma Carcinoma, Non-Cardia Gastric Cancer: NCGC, GCA: Gastric Cardia Adenocarcinoma, BMI: Body mass index, Q.A: quality assessment. UK: united kingdom, USA: the united states of America, SD: standard deviation. | | | | | | | | |

| Supplementary Table 3. The quality assessment scores of the included studies based on NOS | | | | | | | | | |
| --- | --- | --- | --- | --- | --- | --- | --- | --- | --- |
| Study | selection (maximum 4) | | | | comparability (maximum 2) | outcome (maximum 3) | | | Overall^*^ |
|  | representativeness (1) | non-exposed selection (1) | Ascertainment of exposure (1) | no outcome in beginning (1) |  | assessment (1) | follow-up for outcome occurrence (1) | median follow-up (1) |  |
| Aarestrup 2014 (37) | 0 | 1 | 1 | 1 | 2 | 1 | 1 | 1 | 8 |
| Aarestrup 2017 (38) | 0 | 1 | 1 | 1 | 2 | 1 | 1 | 1 | 8 |
| Aarestrup 2016 (39) | 0 | 1 | 1 | 1 | 2 | 1 | 1 | 1 | 8 |
| Aarestrup 2019 (9) | 0 | 1 | 1 | 1 | 2 | 1 | 1 | 1 | 8 |
| Ahlgren 2006 (11) | 0 | 1 | 1 | 1 | 2 | 1 | 1 | 1 | 8 |
| Anderson 2014 (30) | 0 | 1 | 1 | 1 | 2 | 1 | 1 | 1 | 8 |
| T. L. Berentzen 2014 (40) | 0 | 1 | 1 | 1 | 2 | 1 | 1 | 1 | 8 |
| T. Bjørge 2008 (31) | 1 | 0 | 1 | 1 | 2 | 0 | 1 | 1 | 7 |
| T. Bjørge 2004 (41) | 1 | 1 | 1 | 1 | 1 | 0 | 1 | 1 | 7 |
| T. Bjørge 2006 (42) | 1 | 1 | 1 | 1 | 2 | 1 | 1 | 1 | 9 |
| Celind 2020 (7) | 0 | 1 | 1 | 1 | 2 | 1 | 1 | 1 | 8 |
| Celind 2019 (43) | 0 | 1 | 1 | 0 | 2 | 1 | 1 | 1 | 7 |
| Cook 2015 (44) | 0 | 1 | 1 | 1 | 2 | 1 | 1 | 1 | 8 |
| De Stavola 2004 (28) | 1 | 1 | 1 | 1 | 2 | 0 | 1 | 1 | 8 |
| Engeland 2003 (45) | 1 | 1 | 1 | 1 | 2 | 1 | 1 | 1 | 9 |
| Farfel 2014 (12) | 1 | 1 | 1 | 0 | 2 | 1 | 1 | 1 | 8 |
| Furer 2020 (34) | 1 | 1 | 1 | 1 | 2 | 1 | 1 | 1 | 9 |
| Hagström 2018 (46) | 1 | 1 | 1 | 1 | 2 | 1 | 1 | 1 | 9 |
| Jeffreys 2004 (47) | 0 | 1 | 1 | 0 | 2 | 1 | 1 | 1 | 7 |
| Jensen 2018 (48) | 0 | 1 | 1 | 1 | 2 | 1 | 1 | 1 | 8 |
| Jensen 2017 (49) | 0 | 1 | 1 | 1 | 2 | 1 | 1 | 1 | 8 |
| Jensen 2020 (8) | 0 | 1 | 1 | 1 | 2 | 1 | 1 | 1 | 8 |
| Kantor 2016 (50) | 1 | 1 | 1 | 1 | 2 | 1 | 1 | 1 | 9 |
| Katz 2018 (51) | 0 | 1 | 1 | 1 | 2 | 1 | 1 | 1 | 8 |
| Keinan 2020 (52) | 1 | 1 | 1 | 0 | 2 | 1 | 1 | 1 | 8 |
| Keinan 2016 (29) | 1 | 1 | 1 | 1 | 2 | 1 | 1 | 1 | 9 |
| Keinan 2018 (53) | 1 | 1 | 1 | 1 | 2 | 1 | 1 | 1 | 9 |
| Kitahara 2014 (54) | 0 | 1 | 1 | 1 | 2 | 1 | 1 | 1 | 8 |
| Kitahara 2014 (55) | 0 | 1 | 1 | 1 | 2 | 1 | 1 | 1 | 8 |
| Landberg 2019 (10) | 1 | 1 | 1 | 1 | 2 | 1 | 1 | 1 | 9 |
| Leiba 2017 (56) | 1 | 1 | 1 | 1 | 2 | 1 | 1 | 1 | 9 |
| Leiba 2013 (57) | 1 | 1 | 1 | 1 | 2 | 1 | 1 | 1 | 9 |
| Leiba 2012 (58) | 1 | 1 | 1 | 1 | 2 | 1 | 1 | 1 | 9 |
| Leiba 2016 (35) | 1 | 1 | 1 | 0 | 2 | 1 | 1 | 1 | 8 |
| Levi 2012 (59) | 1 | 1 | 1 | 1 | 2 | 1 | 1 | 1 | 9 |
| Levi 2011 (60) | 1 | 1 | 1 | 1 | 1 | 1 | 1 | 1 | 8 |
| Levi 2017 (61) | 1 | 1 | 1 | 1 | 1 | 1 | 1 | 1 | 8 |
| Levi 2013 (36) | 1 | 1 | 1 | 1 | 2 | 1 | 1 | 1 | 9 |
| Levi 2018 (62) | 1 | 1 | 1 | 1 | 1 | 1 | 1 | 1 | 8 |
| Meyle 2017 (63) | 0 | 1 | 1 | 1 | 2 | 1 | 1 | 1 | 8 |
| Nogueira 2017 (64) | 0 | 1 | 1 | 1 | 2 | 1 | 1 | 1 | 8 |
| Nuotio 2021 (65) | 1 | 1 | 1 | 1 | 2 | 1 | 1 | 1 | 9 |
| Petrick 2019 (66) | 0 | 1 | 1 | 1 | 2 | 1 | 1 | 1 | 8 |
| Shamriz 2017 (67) | 1 | 1 | 1 | 1 | 2 | 1 | 1 | 1 | 9 |
| Sørensen 2020 (68) | 0 | 1 | 1 | 1 | 2 | 1 | 1 | 1 | 8 |
| Zohar 2019 (69) | 1 | 1 | 1 | 1 | 2 | 1 | 1 | 1 | 9 |
| NOS: the Newcastle Ottawa scale  ^*^ Based on NOS, the scores for cohort studies range from 0 to 9 | | | | | | | | | |

| Supplementary Table 4. Association of Obesity in minors with malignancies in adulthood in included studies.  ` | | | | | | | | |
| --- | --- | --- | --- | --- | --- | --- | --- | --- |
| First Author (year) | Type of Cancer or Cancer Mortality | Cancer Subgroup | Sex | Type Of DIP (CAT,CONT) | type of effect size | Age range (year) | Effect size (95%CI) | Adjustment |
| Aarestrup 2014  (PRE PSA) (37) | Prostate | (-) | Male | Continuous | HR | 7 | 1.15 (0.97-1.35) | Height |
|  |  |  |  |  |  | 8 | 1.08 (0.91-1.28) |  |
|  |  |  |  |  |  | 9 | 1.08 (0.91-1.29) |  |
|  |  |  |  |  |  | 10 | 1.10 (0.92-1.31) |  |
|  |  |  |  |  |  | 11 | 1.14 (0.96-1.36) |  |
|  |  |  |  |  |  | 12 | 1.14 (0.95-1.37) |  |
|  |  |  |  |  |  | 13 | 1.17 (0.97-1.41) |  |
| Aarestrup 2014  (POST PSA) (37) |  |  |  |  |  | 7 | 1.04 (0.98-1.10) |  |
|  |  |  |  |  |  | 8 | 1.04 (0.98-1.11) |  |
|  |  |  |  |  |  | 9 | 1.02 (0.96-1.09) |  |
|  |  |  |  |  |  | 10 | 1.03 (0.97-1.09) |  |
|  |  |  |  |  |  | 11 | 1.02 (0.96-1.08) |  |
|  |  |  |  |  |  | 12 | 1.02 (0.96-1.08) |  |
|  |  |  |  |  |  | 13 | 1.02 (0.96-1.09) |  |
| Aarestrup 2017 (38) | Endometrial | Estrogen Dependent | Female | Continuous | HR | 7 | 1.19 (1.09-1.31) | Childhood BMI growth |
|  |  | Adenocarcinoma |  |  |  |  | 1.27 (1.13-1.42) |  |
|  |  | Total |  |  |  |  | 1.18 (1.08-1.30) |  |
|  |  | Total |  |  |  | 6.25-7.99 | 1.04 (1.01-1.08) |  |
|  |  |  |  |  |  | 8-10.99 | 1.09 (1.02-1.15) |  |
|  |  |  |  |  |  | 11 to 14 | 1.05 (0.99-1.12) |  |
|  |  |  |  |  |  | total | 1.14 (1.07-1.22) |  |
|  |  | Estrogen Dependent |  |  |  | 6.25-7.99 | 1.03 (0.99-1.07) |  |
|  |  |  |  |  |  | 8-10.99 | 1.08 (1.01-1.14) |  |
|  |  |  |  |  |  | 11 to 14 | 1.04 (0.97-1.11) |  |
|  |  |  |  |  |  | total | 1.11 (1.04-1.2) |  |
|  |  | Adenocarcinoma |  |  |  | 6.25-7.99 | 1.04 (1-1.09) |  |
|  |  |  |  |  |  | 8-10.99 | 1.1 (1.023-1.18) |  |
|  |  |  |  |  |  | 11 to 14 | 1.04 (0.95-1.12) |  |
|  |  |  |  |  |  | total | 1.15 (1.06-1.24) |  |
| Aarestrup 2016 (39) | Endometrial | Non Estrogen Dependent | Female | Continuous | HR | 7 | 1.00 (0.78-1.27) | Height |
|  |  |  |  |  |  | 8 | 1.15 (0.90-1.48) |  |
|  |  |  |  |  |  | 9 | 1.11 (0.86-1.42) |  |
|  |  |  |  |  |  | 10 | 1.20 (0.94-1.55) |  |
|  |  |  |  |  |  | 11 | 1.23 (0.95-1.59) |  |
|  |  |  |  |  |  | 12 | 1.30 (1.01-1.68) |  |
|  |  |  |  |  |  | 13 | 1.31 (1.02-1.68) |  |
|  |  | Adenocarcinoma |  |  |  | 7 | 1.31 (1.16-1.49) | Hormone Use |
|  |  |  |  |  |  | 8 | 1.31 (1.15-1.48) |  |
|  |  |  |  |  |  | 9 | 1.30 (1.14-1.47) |  |
|  |  |  |  |  |  | 10 | 1.39 (1.22-1.58) |  |
|  |  |  |  |  |  | 11 | 1.37 (1.21-1.56) |  |
|  |  |  |  |  |  | 12 | 1.38 (1.21-1.56) |  |
|  |  |  |  |  |  | 13 | 1.38 (1.21-1.58) |  |
| Aarestrup 2019 (9) | Ovaries | Total | Female | Categorical Cutoffs: (BMI 7 Years: 17.69, BMI 8 Years: 18.28, BMI 9 Years: 18 99, BMI 10 Years: 19.78, BMI 11 Years: 20.66, BMI 12 Years: 21.59, BMI 13 Years: 22.49.) | HR | 7 | 1.33 (1.03-1.73) | Birth Weight |
|  |  |  |  |  |  | 8 | 1.24 (0.96-1.60) |  |
|  |  |  |  |  |  | 9 | 1.34 (1.05-1.71) |  |
|  |  |  |  |  |  | 10 | 1.29 (1.01-1.65) |  |
|  |  |  |  |  |  | 11 | 1.27 (0.98-1.64) |  |
|  |  |  |  |  |  | 12 | 1.31 (1.02-1.69) |  |
|  |  |  |  |  |  | 13 | 1.33 (1.05-1.70) |  |
|  |  | Serous |  |  |  | 7 | 0.91 (0.90-1.38) |  |
|  |  |  |  |  |  | 8 | 0.75 (0.48-1.16) |  |
|  |  |  |  |  |  | 9 | 1.02 (0.71-1.48) |  |
|  |  |  |  |  |  | 10 | 1.07 (0.75-1.54) |  |
|  |  |  |  |  |  | 11 | 1.03 (0.70-1.50) |  |
|  |  |  |  |  |  | 12 | 1.09 (0.76-1.58) |  |
|  |  |  |  |  |  | 13 | 0.93 (0.63-1.36) |  |
|  |  | Mucinous |  |  |  | 7 | 2.48 (1.36-4.52) |  |
|  |  |  |  |  |  | 8 | 2.11 (1.13-3.95) |  |
|  |  |  |  |  |  | 9 | 1.97 (1.05-3.69) |  |
|  |  |  |  |  |  | 10 | 2.06 (1.10-3.86) |  |
|  |  |  |  |  |  | 11 | 1.89 (0.98-3.63) |  |
|  |  |  |  |  |  | 12 | 1.68 (0.85-3.34) |  |
|  |  |  |  |  |  | 13 | 1.98 (1.06-3.70) |  |
|  |  | Endometrioid |  |  |  | 7 | 1.76 (0.85-3.62) |  |
|  |  |  |  |  |  | 8 | 1.82 (0.92-3.62) |  |
|  |  |  |  |  |  | 9 | 2.16 (1.15-4.06) |  |
|  |  |  |  |  |  | 10 | 1.78 (0.90-3.55) |  |
|  |  |  |  |  |  | 11 | 2.12 (1.10-4.09) |  |
|  |  |  |  |  |  | 12 | 2.11 (1.09-4.06) |  |
|  |  |  |  |  |  | 13 | 1.96 (1.01-3.77) |  |
|  |  | Clear Cell |  |  |  | 7 | 3.01 (1.17-7.74) |  |
|  |  |  |  |  |  | 8 | 3.41 (1.42-8.19) |  |
|  |  |  |  |  |  | 9 | 3.71 (1.63-8.46) |  |
|  |  |  |  |  |  | 10 | 3.14 (1.31-7.53) |  |
|  |  |  |  |  |  | 11 | 2.09 (0.74-5.90) |  |
|  |  |  |  |  |  | 12 | 3.27 (1.36-7.83) |  |
|  |  |  |  |  |  | 13 | 3.11 (1.30-7.47) |  |
|  |  | Other |  |  |  | 7 | 1.40 (0.80-2.46) |  |
|  |  |  |  |  |  | 8 | 1.48 (0.88-2.51) |  |
|  |  |  |  |  |  | 9 | 1.09 (0.61-1.96) |  |
|  |  |  |  |  |  | 10 | 0.93 (0.49-1.75) |  |
|  |  |  |  |  |  | 11 | 1.07 (0.58-1.96) |  |
|  |  |  |  |  |  | 12 | 0.99 (0.52-1.87) |  |
|  |  |  |  |  |  | 13 | 1.52 (0.91-2.53) |  |
| Ahlgren 2006 (11) | Breast | (-) | Female | Continuous | RR | under 8 | 0.94 (0.91-0.97) | Attained Age and Calendar Period, Age at Peak Growth, and Height at 14 Years |
|  |  |  |  |  |  | 8 to 14 | 0.96 (0.93-0.99) |  |
| Anderson 2014 (30) | Breast | (-) | Female | Continuous | HR | 7 | 0.97 (0.88-1.06) | Age (Underlying Time Scale) and Mammographic Density, and Stratified By Birth Cohort (1930 To 1934, 1935 To 1939, 1940 To 1944, 1945 To 1949) |
|  |  |  |  |  |  | 8 | 1.01 (0.92-1.11) |  |
|  |  |  |  |  |  | 9 | 0.99 (0.90-1.09) |  |
|  |  |  |  |  |  | 10 | 1.01 (0.92-1.10) |  |
|  |  |  |  |  |  | 11 | 1.05 (0.95-1.14) |  |
|  |  |  |  |  |  | 12 | 1.02 (0.93-1.12) |  |
|  |  |  |  |  |  | 13 | 1.01 (0.93-1.11) |  |
| T. L. Berentzen 2014 (40) | Liver | Primary Liver Cancer | Both | Continuous | HR | 7 | 1.19 (1.04-1.38) | Without a Registered Diagnosis of Viral Hepatitis, Alcohol-Related Disorders and Biliary Cirrhosis and Sex and Year Of Birth |
|  |  |  |  |  |  | 8 | 1.21 (1.05-1.41) |  |
|  |  |  |  |  |  | 9 | 1.22 (1.06-1.42) |  |
|  |  |  |  |  |  | 10 | 1.30 (1.12-1.51) |  |
|  |  |  |  |  |  | 11 | 1.26 (1.09-1.46) |  |
|  |  |  |  |  |  | 12 | 1.30 (1.12-1.50) |  |
|  |  |  |  |  |  | 13 | 1.36 (1.17-1.58) |  |
|  |  | HCC |  |  |  | 7 | 1.17 (0.97-1.40) |  |
|  |  |  |  |  |  | 8 | 1.16 (0.96-1.40) |  |
|  |  |  |  |  |  | 9 | 1.15 (0.95-1.40) |  |
|  |  |  |  |  |  | 10 | 1.27 (1.04-1.53) |  |
|  |  |  |  |  |  | 11 | 1.24 (1.02-1.49) |  |
|  |  |  |  |  |  | 12 | 1.31 (1.08-1.58) |  |
|  |  |  |  |  |  | 13 | 1.39 (1.15-1.69) |  |
| T. Bjørge 2008 (31) | Cancer Mortality | Colon | Male | Categorical | RR | 14-19 | 1.0 (0.4-2.2) | Age and Birth Year |
|  |  | Respiratory |  |  |  |  | 0.9 (0.6-1.5) |  |
|  |  | Total |  |  |  |  | 1.0 (0.8-1.2) |  |
|  |  | Hematologic/Lymphatic |  |  |  |  | 0.9 (0.5-1.5) |  |
|  |  | Colon | Female |  |  |  | 1.4 (0.8-2.5) |  |
|  |  | Respiratory |  |  |  |  | 0.7 (0.4-1.2) |  |
|  |  | Hematologic/Lymphatic |  |  |  |  | 1.1 (0.7-1.8) |  |
|  |  | Breast |  |  |  |  | 1.1 (0.8-1.4) |  |
|  |  | Total |  |  |  |  | 1.0 (0.9-1.2) |  |
|  |  | Cervix |  |  |  |  | 0.9 (0.5-1.7) |  |
|  |  | Ovary` |  |  |  |  | 1.0 (0.6-1.7) |  |
|  | Cancer Mortality | Colon | Male |  |  |  | 2.1 (1.1-4.1) |  |
|  |  | Respiratory |  |  |  |  | 1.2 (0.7-2.0) |  |
|  |  | Total |  |  |  |  | 1.2 (0.9-1.5) |  |
|  |  | Hematologic/Lymphatic |  |  |  |  | 1.2 (0.7-2.1) |  |
|  |  | Colon | Female |  |  |  | 2.0 (1.2-3.5) |  |
|  |  | Respiratory |  |  |  |  | 1.1 (0.7-1.8) |  |
|  |  | Hematologic/Lymphatic |  |  |  |  | 1.1 (0.6-1.8) |  |
|  |  | Breast |  |  |  |  | 0.9 (0.6-1.2) |  |
|  |  | Cervix |  |  |  |  | 1.9 (1.1-3.2) |  |
|  |  | Total |  |  |  |  | 1.2 (1.1-1.5) |  |
|  |  | Ovary` |  |  |  |  | 0.6 (0.3-1.4) |  |
| T. Bjørge 2004 (41) | RCC | (-) | Male | Categorical | RR | 14-19 | 1.60 (0.88-2.90) | Age at Measurement (Two Categories) And Birth Cohort (Two Categories) |
|  |  |  | Female |  |  |  | 1.45 (0.63-3.34) |  |
|  |  |  | Male |  |  |  | 2.64 (1.48-4.70) |  |
|  |  |  | Female |  |  |  | 1.48 (0.57-3.85) |  |
| T. Bjørge 2006 (42) | Testicular Cancer | Seminoma | Male | Categorical | RR | 14-19 | 1.29 (0.84-1.98) | Age at Measurement (Two Categories) And Birth Cohort (Two Categories) |
|  |  | Non-Seminoma |  |  |  |  | 0.96 (0.57-1.60) |  |
|  |  | Total |  |  |  |  | 1.15 (0.83-1.59) |  |
|  |  | Seminoma |  |  |  |  | 1.03 (0.58-1.82) |  |
|  |  | Non-Seminoma |  |  |  |  | 0.88 (0.46-1.67) |  |
|  |  | Total |  |  |  |  | 0.95 (0.62-1.45) |  |
| Celind 2020 (7) | Hematologic | (-) | Both | Continuous | HR | 8 | 1.11 (1.02-1.21) | Birth Year, Country of Birth, Height and Pubertal BMI |
| Celind 2019 (43) | GI | Rectum | Both | Continuous | HR | 8 | 0.99 (0.85-1.16) | Height |
|  |  | Colon |  |  |  |  | 1.19 (1.06-1.33) |  |
|  |  | Rectum |  | Categorical Cut Off: (BMI 17.9 ) |  |  | 1.17 (0.63-2.16) |  |
|  |  | Colon |  |  |  |  | 1.78 (1.19-2.67) |  |
| Cook 2015 (44) | GI | Esophagus | Male | Continuous | HR | 7 | 1.11 (0.95-1.30) | Height |
|  |  |  |  |  |  | 8 | 1.10 (0.94-1.29) |  |
|  |  |  |  |  |  | 9 | 1.15 (0.98-1.35) |  |
|  |  |  |  |  |  | 10 | 1.18 (1.00-1.38) |  |
|  |  |  |  |  |  | 11 | 1.21 (1.03-1.42) |  |
|  |  |  |  |  |  | 12 | 1.25 (1.07-1.47) |  |
|  |  |  |  |  |  | 13 | 1.25 (1.06-1.46) |  |
|  |  |  | Female |  |  | 7 | 1.30 (0.90-1.87) |  |
|  |  |  |  |  |  | 8 | 1.41 (0.97-2.06) |  |
|  |  |  |  |  |  | 9 | 1.49 (1.02-2.16) |  |
|  |  |  |  |  |  | 10 | 1.44 (0.99-2.11) |  |
|  |  |  |  |  |  | 11 | 1.63 (1.12-2.36) |  |
|  |  |  |  |  |  | 12 | 1.55 (1.07-2.26) |  |
|  |  |  |  |  |  | 13 | 1.68 (1.15-2.44) |  |
|  |  |  | Total |  |  | 7 | 1.14 (0.99-1.31) |  |
|  |  |  |  |  |  | 8 | 1.14 (0.99-1.32) |  |
|  |  |  |  |  |  | 9 | 1.20 (1.03-1.39) |  |
|  |  |  |  |  |  | 10 | 1.21 (1.05-1.41) |  |
|  |  |  |  |  |  | 11 | 1.26 (1.09-1.47) |  |
|  |  |  |  |  |  | 12 | 1.30 (1.12-1.50) |  |
|  |  |  |  |  |  | 13 | 1.31 (1.13-1.51) |  |
| De Stavola 2004 (28) | Breast | (-) | Female | Continuous | OR | 2 | 1.02 (0.78-1.33) | Anthropometric variables |
|  |  |  |  |  |  | 4 | 0.88 (0.67-1.14) |  |
|  |  |  |  |  |  | 7 | 0.87 (0.66-1.15) |  |
|  |  |  |  |  |  | 11 | 0.89 (0.68-1.18) |  |
|  |  |  |  |  |  | 15 | 0.86 (0.65-1.14) |  |
| Engeland 2003 (45) | Ovaries | (-) | Female | Categorical | RR | 14 to 19 | 1.43 (1.00-2.04) | Age at Measurement and Birth Cohort |
|  |  |  |  |  |  |  | 1.56 (1.04-2.32) |  |
| Farfel 2014 (12) | Thyroid | (-) | Male | Categorical 4th Quintile 21.8–24.01 | HR | 16 to 19 | 1.12 (0.80-1.58) | Year of Birth |
|  |  |  | Female | Categorical 4th Quintile 21.67–23.62 |  |  | 1.04 (0.76-1.42) |  |
|  |  |  | Male | Categorical 5th Quintile > 24.01 Kg/M |  |  | 1.14 (0.81-1.60) |  |
|  |  |  | Female | Categorical 5th Quintile > 26.62 Kg/M |  |  | 1.19 (0.87-1.63) |  |
| Furer 2020 (34) | Various | Total | Male | Categorical  percentile 50th–74th | HR | 16 to 19 | 1.03 (1.01-1.06) | Age at Study Entry, Birth Year, Region of Origin, Residential Socioeconomic Status, and Education Minus Cervix and Brest Cancer |
|  |  |  | Female |  |  |  | 1.10 (1.06-1.14) |  |
|  |  |  | Male | Categorical  percentile 75th–84th |  |  | 1.12 (1.07-1.17) |  |
|  |  |  | Female |  |  |  | 1.15 (1.09-1.21) |  |
|  |  |  | Male | Categorical  percentile 85th–94th |  |  | 1.17 (1.12-1.23) |  |
|  |  |  | Female |  |  |  | 1.21 (1.14-1.28) |  |
|  |  |  | Male | Categorical  percentile ≥95th |  |  | 1.26 (1.26-1.35) |  |
|  |  |  | Female |  |  |  | 1.27 (1.13-1.44) |  |
|  |  |  | Both First Cohort | Categorical  percentile 50th–84th |  |  | 1.05 (1.00-1.11) | Age at Study Entry, Sex, Birth Year, Residential Socioeconomic Status,  Country of Origin, and Education |
|  |  |  | Both Second Cohort |  |  |  | 1.23 (1.17-1.29) |  |
|  |  |  | Both First Cohort | Categorical  percentile ≥85th |  |  | 1.13 (1.04-1.24) |  |
|  |  |  | Both Second Cohort |  |  |  | 1.36 (1.27-1.45) |  |
|  |  | Brain | Both First Cohort | Categorical increase in BMI (Per 5 Kg/M²) |  |  | 1.01 (0.86-1.18) |  |
|  |  | Brain | Both Second Cohort | Categorical increase in BMI (Per 5 Kg/M²) |  |  | 1.06 (0.95-1.18) |  |
|  |  | Thyroid | Both First Cohort | Categorical increase in BMI (Per 5 Kg/M²) |  |  | 1.01 (0.85-1.20) |  |
|  |  | Thyroid | Both Second Cohort | Categorical increase in BMI (Per 5 Kg/M²) |  |  | 1.12 ( 1.04-1.21) |  |
|  |  | Non Hodgkin Lymphoma | Both First Cohort | Categorical increase in BMI (Per 5 Kg/M²) |  |  | 1.06 (0.91-1.22) |  |
|  |  | Non Hodgkin Lymphoma | Both Second Cohort | Categorical increase in BMI (Per 5 Kg/M²) |  |  | 1.15 (1.05-1.26) |  |
|  |  | Colorectal Cancer | Both First Cohort | Categorical increase in BMI (Per 5 Kg/M²) |  |  | 1.12 (0.93-1.36) |  |
|  |  | Colorectal Cancer | Both Second Cohort | Categorical increase in BMI (Per 5 Kg/M²) |  |  | 1.19 (1.04-1.36) |  |
|  |  | Oral Cavity Cancer | Both First Cohort | Categorical increase in BMI (Per 5 Kg/M²) |  |  | 1.15 (0.95-1.38) |  |
|  |  | Oral Cavity Cancer | Both Second Cohort | Categorical increase in BMI (Per 5 Kg/M²) |  |  | 1.34 (1.11-1.63) |  |
|  |  | Stomach Cancer | Both First Cohort | Categorical increase in BMI (Per 5 Kg/M²) |  |  | 1.03 (0.97-1.10) |  |
|  |  | Stomach Cancer | Both Second Cohort | Categorical increase in BMI (Per 5 Kg/M²) |  |  | 1.08 (1.02-1.12) |  |
|  |  | Hodgkin Lymphoma | Both First Cohort | Categorical increase in BMI (Per 5 Kg/M²) |  |  | 1.21 (1.03-1.42) |  |
|  |  | Hodgkin Lymphoma | Both Second Cohort | Categorical increase in BMI (Per 5 Kg/M²) |  |  | 1.15 (1.05-1.26) |  |
|  |  | Leukemia | Both First Cohort | Categorical increase in BMI (Per 5 Kg/M²) |  |  | 1.16 (0.94-1.44) |  |
|  |  | Leukemia | Both Second Cohort | Categorical increase in BMI (Per 5 Kg/M²) |  |  | 1.08 (0.94-1.27) |  |
|  |  | Cervical Cancer | Both First Cohort | Categorical increase in BMI (Per 5 Kg/M²) |  |  | 0.95 (0.83-1.09) |  |
|  |  | Cervical Cancer | Both Second Cohort | Categorical increase in BMI (Per 5 Kg/M²) |  |  | 0.89 (0.84-0.94) |  |
|  |  | Ovarian Cancer | Female First Cohort | Categorical increase in BMI (Per 5 Kg/M²) |  |  | 1.50 (1.13-2.01) |  |
|  |  | Ovarian Cancer | Female Second Cohort | Categorical increase in BMI (Per 5 Kg/M²) |  |  | 1.17 (0.93-1.47) |  |
|  |  | Testis Cancer | Male First Cohort | Categorical increase in BMI (Per 5 Kg/M²) |  |  | 1.08 (0.90-1.29) |  |
|  |  | Testis Cancer | Male Second Cohort | Categorical increase in BMI (Per 5 Kg/M²) |  |  | 1.02 (0.91-1.13) |  |
|  |  | Breast Cancer | Female First Cohort | Categorical increase in BMI (Per 5 Kg/M²) |  |  | 0.88 (0.80-0.98) |  |
|  |  | Breast Cancer | Female Second Cohort | Categorical increase in BMI (Per 5 Kg/M²) |  |  | 0.88 (0.83-0.94) |  |
|  |  | Melanoma | Both First Cohort | Categorical increase in BMI (Per 5 Kg/M²) |  |  | 0.97 (0.88-1.08) |  |
|  |  | Melanoma | Both Second Cohort | Categorical increase in BMI (Per 5 Kg/M²) |  |  | 0.98 (0.91-1.06) |  |
|  | Cancer Survival | Total | Male | Categorical  percentile 50th–74th |  |  | 0.97 (0.92-1.03) | Age at Cancer Diagnosis, Birth Year, Region of Origin, Residential  Socioeconomic Status, and Education. |
|  |  |  | Female |  |  |  | 1.07 (1.01-1.15) |  |
|  |  |  | Male | Categorical  percentile 75th–84th |  |  | 1.04 (0.96-1.13) |  |
|  |  |  | Female |  |  |  | 1.14 (1.04-1.25) |  |
|  |  |  | Male | Categorical  percentile 85th–94th |  |  | 1.11 (1.02-1.21) |  |
|  |  |  | Female |  |  |  | 1.26 (1.14-1.40) |  |
|  |  |  | Male | Categorical  percentile ≥95th |  |  | 1.33 (1.18-1.49) |  |
|  |  |  | Female |  |  |  | 1.89 (1.56-2.28) |  |
| Hagström 2018 (46) | HCC | (-) | Male | Categorical 22.5≤BMI<25 | HR | 17 to 19 | 1.28 (0.91-1.80) | Age and Year of Birth, Location of Conscription, Own And Parental Education And Parental Socioeconomic Status, Scores on Intelligence Test, Cardiovascular Capacity and Muscular Strength Tests, Systolic And Diastolic Blood Pressures. |
|  |  |  |  | Categorical 25≤BMI<30 |  |  | 1.57 (1.01-2.47) |  |
|  |  |  |  | Categorical BMI≥30 |  |  | 3.59 (1.85-6.99) |  |
| Jeffreys 2004 (47) | Colorectal | (-) | Both | Continuous | OR | 2 to 14 | 0.96 (0.70-1.32) | Age and Sex |
|  | Prostate | (-) | Male |  |  |  | 1.12 (0.72-1.72) |  |
|  | Breast | (-) | Female |  |  |  | 0.95 (0.73-1.25) |  |
|  | Lung | (-) | Both |  |  |  | 1.27 (1.03-1.57) |  |
| Jensen 2018 (48) | Colon | (-) | Male | (7 Years BMI ≥ 17.88 Kg/M2, 13 Years BMI ≥ 21.89, 17 Years BMI ≥ 24.46–24.96 Kg/M2) | HR | 7 to 26 (7 to 19) | 2.62 (1.62-4.25) | Educational Level and Age at The Conscription Examination Minimally Influenced The Results |
| Jensen 2017 (49) | Non-Sigmoid Colon | (-) | Male | Continuous | HR | 7 | 1.06 (0.98-1.15) | Sex and Birth Cohort |
|  |  |  |  |  |  | 10 | 1.05 (0.97-1.15) |  |
|  |  |  |  |  |  | 13 | 1.06 (0.97-1.16) |  |
|  | Sigmoid Colon | (-) |  |  |  | 7 | 1.05 (0.96-1.15) |  |
|  |  |  |  |  |  | 10 | 1.11 (1.01-1.22) |  |
|  |  |  |  |  |  | 13 | 1.14 (1.03-1.25) |  |
|  | Non-Sigmoid Colon | (-) | Female |  |  | 7 | 1.07 (0.98-1.16) |  |
|  |  |  |  |  |  | 10 | 1.10 (1.01-1.20) |  |
|  |  |  |  |  |  | 13 | 1.08 (0.99-1.18) |  |
|  | Sigmoid Colon | (-) |  |  |  | 7 | 1.10 (1.00-1.21) |  |
|  |  |  |  |  |  | 10 | 1.11 (1.00-1.23) |  |
|  |  |  |  |  |  | 13 | 1.08 (0.98-1.19) |  |
|  | Non-Sigmoid Colon | (-) | Both |  |  | 7 | 1.06 (1.00-1.13) |  |
|  |  |  |  |  |  | 10 | 1.08 (1.01-1.14) |  |
|  |  |  |  |  |  | 13 | 1.07 (1.01-1.14) |  |
|  | Sigmoid Colon | (-) |  |  |  | 7 | 1.07 (1.00-1.15) |  |
|  |  |  |  |  |  | 10 | 1.11 (1.03-1.19) |  |
|  |  |  |  |  |  | 13 | 1.11 (1.04-1.19) |  |
| Jensen 2020 (8) | Renal Cell Carcinoma | (-) | Both | Categorical Girls: Age 7 Years: BMI ≥ 17.69 Kg/M2; Age 10 Years: BMI ≥ 19.78 Kg/M2; Age 13 Years: BMI ≥ 22.49 Kg/M2. Boys: Age 7 Years: BMI ≥ 17.88 Kg/M2; Age 10 Years: BMI ≥ 19.80 Kg/M2; Age 13 Years: BMI ≥ 21.89 Kg/M2 | HR | 7 | 1.03 (0.73-1.47) | Height or Birth Weight |
|  |  |  |  |  |  | 10 | 1.15 (0.85-1.55) |  |
|  |  |  |  |  |  | 13 | 1.40 (1.08-1.81) |  |
| Kantor 2016 (50) | Colorectal Cancer | (-) | Males | Normal Weight (BMI:18.5<25), Lower Overweight (BMI:25-<27.5), Upper Overweight (BMI:27.5-<30), and Obese (BMI:30+; Ranging From 30-<55). | HR | 16 to 20 | 1.12 (0.84-1.50) | Age at Conscription |
|  |  |  |  |  |  |  | 2.03 (1.38-2.98) |  |
|  |  |  |  |  |  |  | 2.38 (1.53-3.72) |  |
| Katz 2018 (51) | Gep-Net | (-) | Both | Categorical (CDC based Age-Sex Specific BMI) | HR | 16 to 19 | 2.43 (1.21-4.86) | Year of examination, sex and suspected confounders that showed an association in the univariate analysis at the p < 0.10 |
| Keinan 2020 (52) | Cervix | (-) | Female | 25.0–29.9=Overweight, and ≥30.0=Obese | HR | 17 | 1.19 (0.96-1.48) | Lesion Type,  Country of Origin, Measured Body Mass Index, Height, Education,  Dwelling Type, Birth Year, And Age at Examination. |
|  |  |  |  |  |  |  | 1.10 (0.66-1.85) |  |
| Keinan 2016 (29) | Breast | (-) | Female | Weight; 25.0–29.9: Overweight; and C30.0: Obese | HR | 16-19 | 0.94 (0.83-1.07) | Country of Origin, Education, and Height |
|  |  |  |  |  |  |  | 0.69 (0.48-1.00) |  |
| Keinan 2018 (53) | Breast | Male Breast Cancer | Male | Overweight (25.0-29.9 Kg/M2), And Obese (>30.0 Kg/M2); | HR | 17 | 2.01 (1.14-3.54) | Year of Birth, Country of Origin and General Intelligence Test Score, |
|  |  |  |  |  |  |  | 4.97 (2.14-11.53) |  |
| Kitahara 2014 (54) | Thyroid | (-) | Male | Continuous | HR | 7 | 1.22 (0.93-1.60) | Height SDS  and Birth Weight SDS, |
|  |  |  |  |  |  | 8 | 1.24 (0.94-1.63) |  |
|  |  |  |  |  |  | 9 | 1.23 (0.93-1.63) |  |
|  |  |  |  |  |  | 10 | 1.21 (0.91-1.60) |  |
|  |  |  |  |  |  | 11 | 1.24 (0.94-1.65) |  |
|  |  |  |  |  |  | 12 | 1.25 (0.94-1.66) |  |
|  |  |  |  |  |  | 13 | 1.25 (0.93-1.66) |  |
|  |  |  | Female |  |  | 7 | 1.13 (0.96-1.33) |  |
|  |  |  |  |  |  | 8 | 1.12 (0.95-1.32) |  |
|  |  |  |  |  |  | 9 | 1.18 (1.00-1.39) |  |
|  |  |  |  |  |  | 10 | 1.14 (0.96-1.35) |  |
|  |  |  |  |  |  | 11 | 1.11 (0.94-1.31) |  |
|  |  |  |  |  |  | 12 | 1.09 (0.92-1.29) |  |
|  |  |  |  |  |  | 13 | 1.13 (0.96-1.34) |  |
|  | Thyroid | Papillary | Both |  |  | 7 | 1.21 (1.01-1.43) |  |
|  |  |  |  |  |  | 8 | 1.25 (1.05-1.49) |  |
|  |  |  |  |  |  | 9 | 1.30 (1.08-1.55) |  |
|  |  |  |  |  |  | 10 | 1.27 (1.06-1.53) |  |
|  |  |  |  |  |  | 11 | 1.26 (1.05-1.51) |  |
|  |  |  |  |  |  | 12 | 1.24 (1.03-1.49) |  |
|  |  |  |  |  |  | 13 | 1.28 (1.06-1.53) |  |
|  |  | Follicular |  |  |  | 7 | 1.21 (0.92-1.61) |  |
|  |  |  |  |  |  | 8 | 1.06 (0.80-1.42) |  |
|  |  |  |  |  |  | 9 | 1.09 (0.82-1.46) |  |
|  |  |  |  |  |  | 10 | 1.05 (0.78-1.41) |  |
|  |  |  |  |  |  | 11 | 1.00 (0.75-1.35) |  |
|  |  |  |  |  |  | 12 | 0.99 (0.74-1.33) |  |
|  |  |  |  |  |  | 13 | 1.00 (0.74-1.34) |  |
| Kitahara 2014 (55) | Glioblastoma | (-) | Male | Continuous | HR | 7 | 1.01 (0.86-1.17) | Height SDS  and Birth Weight SDS, |
|  |  |  |  |  |  | 8 | 1.04 (0.89-1.22) |  |
|  |  |  |  |  |  | 9 | 1.03 (0.88-1.21) |  |
|  |  |  |  |  |  | 10 | 1.02 (0.87-1.19) |  |
|  |  |  |  |  |  | 11 | 1.02 (0.87-1.19) |  |
|  |  |  |  |  |  | 12 | 1.00 (0.86-1.17) |  |
|  |  |  |  |  |  | 13 | 1.04 (0.89-1.21) |  |
|  |  |  | Female |  |  | 7 | 0.96 (0.79-1.16) |  |
|  |  |  |  |  |  | 8 | 0.95 (0.79-1.16) |  |
|  |  |  |  |  |  | 9 | 0.95 (0.79-1.16) |  |
|  |  |  |  |  |  | 10 | 0.87 (0.72-1.06) |  |
|  |  |  |  |  |  | 11 | 0.93 (0.76-1.13) |  |
|  |  |  |  |  |  | 12 | 0.91 (0.75-1.10) |  |
|  |  |  |  |  |  | 13 | 1.01 (0.83-1.22) |  |
| Landberg 2019 (10) | Renal Cell Carcinoma | (-) | Male | Categorical Overweight (25 To <30), Obese (≥ 30) | HR | 16 to 20 | 1.76 (1.16-2.67) | Age at Conscription, Household Crowding, Parental SEI, Systolic Blood Pressure, Diastolic Blood Pressure, Height, Health Status at Conscription, ESR, Erythrocyte Volume Fraction, Muscular Strength and Cognitive Function |
|  |  |  |  |  |  |  | 2.87 (1.32-6.25) |  |
|  |  |  |  | Continuous |  |  | 1.06 (1.01-1.11) |  |
| Leiba 2017 (56) | Hematologic | Myeloproliferative | Both | Categorical  percentile 50th–84th | HR | 16 to 19 | 0.89 (0.60-1.32) | Sex |
|  |  |  |  | Categorical  percentile ≥85th |  |  | 1.81 (1.13-2.92) |  |
| Leiba 2013 (57) | Renal Cell Carcinoma | (-) | Male | Categorical  percentile 22.5–24.9 | HR | 16 to 20 | 1.28 (0.96-1.73) | Birth Year, BMI (Less Than 22.5, 22.5 To 24.9, 25.0 To 27.4 and 27.5 Kg/M2 or Greater Dummy Variable), Height (Quintiles, Dummy Variable) and Ethnic Origin (4 Groups, Dummy Variable). |
|  |  |  |  | Categorical  percentile 25.0–27.4 |  |  | 1.16 (0.72-1.87) |  |
|  |  |  |  | Categorical  percentile 27.5 or Greater |  |  | 2.43 (1.54-3.83) |  |
| Leiba 2012 (58) | Urothelial Cancer | (-) | Both | Categorical 85th Percentile | HR | 16 to 19 | 1.42 (1.13-1.77) | Year of Birth, BMI, Years of Education and School Type |
| Leiba 2016 (35) | Non-Hodgkin Lymphoma | 9 | Both | Categorical 85th Percentile | HR | 16 to 19 | 1.25 (1.13-1.37) | Year of Birth, Age at Examination, and Sex (Including Mutual Adjustments For BMI and Height) |
|  |  |  |  | Continuous |  |  | 1.16 (1.10-1.21) |  |
| Levi 2012 (59) | Pancreas | (-) | Both | Categorical  Percentile ≥85th | HR | 16 to 19 | 2.09 (1.25-3.50) | Year of Birth and Years of Schooling |
|  |  |  |  | Continuous |  |  | 2.28 (1.42-3.64) |  |
| Levi 2011 (60) | GI | Colon | Male | Categorical 85th Percentile | HR | 16 to 19 | 1.75 (1.33-2.30) | Year of Birth, Age at BMI Measurement, Country of Origin, Residence (Rural or Urban), Immigration Status, Socioeconomic Status, and Height. |
|  |  | Rectum |  |  |  |  | 1.14 (0.70-1.87) |  |
|  |  | Nonmucinous Colorectal |  |  |  |  | 1.66 (1.29-2.14) |  |
|  |  | Non-mucinous Colon |  |  |  |  | 1.86 ( 1.38-2.50) |  |
|  |  | Mucinous Colorectal |  |  |  |  | 1.02 (0.49-2.13) |  |
| Levi 2017 (61) | GI | Colon | Male | Categorical  BMI: 25 To 29.9 (Overweight), and 30 Kg/M2 (Obese). | HR | 16 to 19 | 1.54 (1.28-1.84) | Birth Year, Age at BMI Measurement, Sex, Socioeconomic Status, and Birth Country |
|  |  |  |  |  |  |  | 1.52 (1.13-2.04) |  |
|  |  |  | Female |  |  |  | 1.55 (1.23-1.94) |  |
|  |  |  |  |  |  |  | 1.52 (0.90-2.59) |  |
|  |  |  | Both |  |  |  | 1.53 (1.34-1.78) |  |
|  |  |  |  |  |  |  | 1.53 (1.18-1.98) |  |
|  |  | Rectum | Male |  |  |  | 1.10 (0.79-1.54) |  |
|  |  |  |  |  |  |  | 1.73 (1.12-2.69) |  |
|  |  |  | Female |  |  |  | 0.85 (0.50-1.44) |  |
|  |  |  |  |  |  |  | 2.07 (0.92-4.66) |  |
|  |  |  | Both |  |  |  | 1.02 (0.77-1.35) |  |
|  |  |  |  |  |  |  | 1.78 (1.21-2.62) |  |
| Levi 2013 (36) | GI | EAC | Male | Categorical 85th Percentile | HR | 16 to 19 | 1.52 (0.52-4.41) | Year of Birth, BMI, Socioeconomic Status, Country of Birth, and Years of Education. |
|  |  | GEJAC |  |  |  |  | 2.91 (1.15-7.37) |  |
|  |  | EAC And GEJAC |  |  |  |  | 2.14 (1.07-4.20) |  |
|  |  | NCGC |  |  |  |  | 1.23 (0.72-2.10) |  |
|  |  | NCGC Intestina |  |  |  |  | 1.40 (0.47-2.73) |  |
|  |  | NCGC Mucinous |  |  |  |  | 0.99 (0.39-2.34) |  |
| Levi 2018 (62) | NCGC | (-) | Both | Categorical  85th To <95th Percentile (Overweight), and 95th Per Centile (Obese) | HR | 16 to 19 | 1.07 (0.77-1.50) | Centers For Disease Control and Prevention Body Mass Index Classification, Residential Socioeconomic Status, Birth Country, Education, Birth Year, and Sex (Only For The Combined Analysis of Men and Women). |
|  |  |  |  |  |  |  | 1.78 (1.12-2.83) |  |
|  |  |  | Male |  |  |  | 1.31 (0.90-1.90) |  |
|  |  |  |  |  |  |  | 1.61 (0.94-2.76) |  |
|  |  |  | Female |  |  |  | 0.55 (0.24-1.24) |  |
|  |  |  |  |  |  |  | 2.55 (1.04-6.26) |  |
| Meyle 2017 (63) | Melanoma | (-) | Both | Continuous | HR | 7 | 1.01 (0.96-1.05) | Childhood Height |
|  |  |  |  |  |  | 8 | 1.00 (0.96-1.05) |  |
|  |  |  |  |  |  | 9 | 0.99 (0.95-1.04) |  |
|  |  |  |  |  |  | 10 | 0.99 (0.94-1.04) |  |
|  |  |  |  |  |  | 11 | 0.97 (0.93-1.02) |  |
|  |  |  |  |  |  | 12 | 0.97 (0.92-1.02) |  |
|  |  |  |  |  |  | 13 | 0.96 (0.91-1.00) |  |
| Nogueira 2017 (64) | Pancreatic Cancer | (-) | Both | Categorical (Cutoff 1.5 Z Score) | HR | never | 1 | Height |
|  |  |  |  |  |  | 7 | 1.32 (0.82-2.13) |  |
|  |  |  |  |  |  | 10 | 1.51 (0.83-2.75) |  |
|  |  |  |  |  |  | 13 | 1.54 (1.00-2.37) |  |
|  |  |  |  |  |  | 7 and 10 | 1.04 (0.46-2.32) |  |
|  |  |  |  |  |  | 7 and 13 | 1.20 (0.39-3.72) |  |
|  |  |  |  |  |  | 10 and 13 | 1.34 (0.78-2.32) |  |
|  |  |  |  |  |  | 7, 10 and 13 | 1.70 (1.13-2.56) |  |
|  |  |  |  |  |  | never | 1 |  |
|  |  |  |  |  |  | 7 | 0.93 (0.30-2.91) |  |
|  |  |  |  |  |  | 10 | 1.52 (0.38-6.12) |  |
|  |  |  |  |  |  | 13 | 1.50 (0.56-4.03) |  |
|  |  |  |  |  |  | 7 and 10 | 1.05 (0.15-7.46) |  |
|  |  |  |  |  |  | 7 and 13 | (-) |  |
|  |  |  |  |  |  | 10 and 13 | (-) |  |
|  |  |  |  |  |  | 7, 10 and 13 | 0.47 (0.67-3.33) |  |
|  |  |  |  |  |  | 7 | 1.03 (0.96-1.10) |  |
|  |  |  |  |  |  | 8 | 1.04 (0.97-1.11) |  |
|  |  |  |  |  |  | 9 | 1.04 (0.97-1.11) |  |
|  |  |  |  |  |  | 10 | 1.03 (0.97-1.10) |  |
|  |  |  |  |  |  | 11 | 1.04 (0.98-1.11) |  |
|  |  |  |  |  |  | 12 | 1.04 (0.97-1.11) |  |
|  |  |  |  |  |  | 13 | 1.05 (0.99-1.13) |  |
|  |  |  |  |  |  | 7 | 1.10 (0.97-1.24) |  |
|  |  |  |  |  |  | 8 | 1.10 (0.97-1.24) |  |
|  |  |  |  |  |  | 9 | 1.11 (0.98-1.25) |  |
|  |  |  |  |  |  | 10 | 1.10 (0.97-1.24) |  |
|  |  |  |  |  |  | 11 | 1.09 (0.96-1.22) |  |
|  |  |  |  |  |  | 12 | 1.07 (0.95-1.21) |  |
|  |  |  |  |  |  | 13 | 1.09 (0.96-1.23) |  |
| Nuotio 2021 (65) | Cancer Mortality | (-) | Both | Continuous | HR | 3 to 19 | 1.24 (1.03-1.49) | Age ,Sex and Study Cohort. |
|  |  |  |  |  |  |  | 0.99 (0.63-1.55) |  |
|  |  |  |  |  |  |  | 1.53 (1.11-2.13) |  |
|  |  |  |  |  |  |  | 1.11 (1.00-1.24) |  |
| Petrick 2019 (66) | GI | EAC | Both | 21.89 Kg/M2 For Boys at Age 13 Years, and 17.88 Kg/M2 For Boys at Age 7 Years, 24.46–24.96 Kg/M2 For Young Men Between 17 and 18 Years (Utilizing Month-Specific Cut-Points). | HR | 7 | 3.11 (1.68-5.76) | Education Level and Age at Conscription |
|  |  | GCA |  |  |  | 7 | 1.55 (0.63-3.79) |  |
|  |  | Total |  |  |  | 7 | 2.36 (1.42-3.92) |  |
|  |  | EAC |  |  |  | 13 | 1.88 (1.06-3.33) |  |
|  |  | GCA |  |  |  | 13 | 1.64 (0.86-3.13) |  |
|  |  | Total |  |  |  | 13 | 1.77 (1.15-2.71) |  |
|  |  | EAC |  |  |  | 18 | 2.11 (1.33-3.33) |  |
|  |  | GCA |  |  |  | 18 | 0.91 (0.46-1.79) |  |
|  |  | Total |  |  |  | 18 | 1.52 (1.05-2.22) |  |
|  |  | Total |  |  |  | never overweight | 1 |  |
|  |  |  |  |  |  | began at age 7 | (-) |  |
|  |  |  |  |  |  | began at age 13 | 1.97 (0.97-3.99) |  |
|  |  |  |  |  |  | began at age 18 | 1.25 (0.73-2.15) |  |
|  |  |  |  |  |  | between 7 to 13 | (-) |  |
|  |  |  |  |  |  | between 7 to 18 | (-) |  |
|  |  |  |  |  |  | between 13 to 18 | 1.38 (0.61-3.11) |  |
|  |  |  |  |  |  | persistently obese | 3.18 (1.57-6.44) |  |
| Shamriz 2017 (67) | Acute Myeloid Leukemia | (-) | Both | Continuous | HR | 16 to 19 | 1.04 (1.01-1.06) | Birth Year, Sex, Country of Origin, and Height |
| Sørensen 2020 (68) | Bladder Cancer | (-) | Both | Continuous | HR | 7 | 1.03 (0.95-1.10) | Sex and Birth Cohort |
|  |  |  |  |  |  | 8 | 1.04 (0.96-1.12) |  |
|  |  |  |  |  |  | 9 | 1.07 (1.00-1.14) |  |
|  |  |  |  |  |  | 10 | 1.11 (1.03-1.18) |  |
|  |  |  |  |  |  | 11 | 1.12 (1.01-1.19) |  |
|  |  |  |  |  |  | 12 | 1.11 (1.04-1.19) |  |
|  |  |  |  |  |  | 13 | 1.10 (1.02-1.17) |  |
| Zohar 2019 (69) | Pancreatic Cancer | (-) | Both | Categorical 5th To <85th Percentile (“Normal” Weight Reference Group), 85th To <95th Percentile (Overweight), and 95th Percentile Or Higher (Obese). | HR | 16 to 19 | 1.68 (1.27-2.23) | Education, Year of Birth, and Sex (Only For The Combined Analysis of Men and Women). |
|  |  |  |  |  |  |  | 3.75 (2.66-5.28) |  |
|  |  |  | Male |  |  |  | 1.87 (1.37-2.56) |  |
|  |  |  |  |  |  |  | 3.69 (2.53-5.36) |  |
|  |  |  | Female |  |  |  | 1.18 (0.63-2.19) |  |
|  |  |  |  |  |  |  | 3.90 (1.70-8.92) |  |
| HCC: hepatocellular carcinoma, RCC: renal cell carcinoma, GI: gastrointestinal, Gep-Net: Gastroenteropancreatic Neuroendocrine Tumors, EAC: Esophageal Adenocarcinoma, GEJAC: Gastroesophageal Junction Adenoma Carcinoma, Non-Cardia Gastric Cancer: NCGC, GCA: Gastric Cardia Adenocarcinoma, BMI: Body mass index, HR: hazard ratio Or: odds ratio, RR: relative risk, SDS: standard deviation score | | | | | | | | |
